# Synthesis of 9-O-arylated berberines via copper-catalyzed C_Ar_–O coupling reactions

**DOI:** 10.3762/bjoc.15.161

**Published:** 2019-07-15

**Authors:** Qiaoqiao Teng, Xinhui Zhu, Qianqian Guo, Weihua Jiang, Jiang Liu, Qi Meng

**Affiliations:** 1 Jiangsu Key Laboratory of Advanced Catalytic Materials and Technology, School of Petrochemical Engineering, Changzhou University, Changzhou 213164, China

**Keywords:** arylation, berberines, cross-coupling, copper, lipophilicity, structural modification

## Abstract

Berberine is a widely used antimicrobial agent in clinic. However, a high dosage is often required due to its low lipophilicity and bioavailability. The current study explores the structural modifications of berberines with potentially lipophilic aryl groups to address this problem. A series of 15 9-O-aryl-substituted berberines (**3a**–**o**) and one 9-O-phenylene-bridged berberine dimer (**5**) was synthesized by copper-catalyzed cross-coupling of tetrahydroberberrubine and aryl iodides, followed by oxidation with I_2_.

## Introduction

Berberine (BBR) is a natural alkaloid extracted from ranunculaceae, rutaceae and berberidaceae, which features a tetracyclic isoquinoline core unit. It was widely used as antibacterial agent [1] and has shown myriad therapeutic potencies in tumor cell suppression [2–3] and various chronic disease management [4]. In most cases, the ammonium motif was identified as the key functioning group. However, it leads to lipophobicity of BBR, making it poorly absorbable. Therefore, high oral doses of BBR are often required for an effective treatment, which is not only uneconomic, but may cause adverse effects as well in long term treatment.

**Scheme 1:**
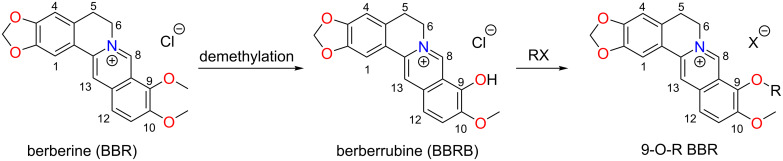
Synthetic pathway of 9-O-R BBR.

The bioavailability, however, could be significantly enhanced through introduction of lipophilic substituents. In this respect, structural modifications with highly lipophilic groups such as long-chain alkyls at C8 [5–6], C9 [7], and C13 [8–9] have been investigated, which all led to considerable improvements in terms of biological activity (Scheme 1, BBR structure). Among all lipophilicity-enhancing strategies, C9-O-Me substitution is the most intensively studied one, which is primarily due to its synthetic convenience. The synthesis starts with pyrolysis of BBR under vacuum at high temperature, during which 9-O-methyl cleavage occurs. The resulting berberrubine (BBRB) then acts as a good precursor to 9-O-substituted derivatives through nucleophilic substitution reactions with alkyl halides (Scheme 1). By this route, not only long-chain alkyl substituents were installed, but also more elaborate motifs such as heterocycles [10–12], glucose [13], nitric oxide [14], and the multidrug-resistance pump inhibitor 5-nitro-2-phenylindole (INF_55_) [15] have been covalently attached to BBR, further widening its pharmacological spectrum.

Aromatic rings are another family of substituents having high lipophilicity, however, they have been rarely used to functionalize BBR. To the best of our knowledge, only three groups have reported the coupling of aryl groups to the C8 [5,16] and the C12 position [17] of BBR, while 9-O-arylation has not been reported thus far. This is quite surprising given the fact that 9-O-functionalization is well-established. Therefore, to further diversify the BBR structure and to provide more potent drug candidates in the future, we herein report the first synthetic study on 9-O-arylated berberine.

## Results and Discussion

**Scheme 2:**
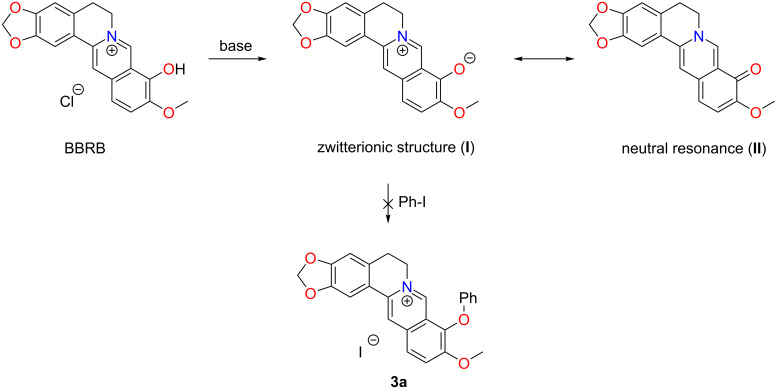
Resonance of berberrubine leading to the failure of direct BBRB cross coupling.

In an analogous manner as described for 9-O-alkylated BBR, the synthesis of 9-O-phenyl BBR **3a** was initially attempted by direct cross-coupling of berberrubine (BBRB) with phenyl iodide, using CuI and 1,10-phenanthroline as the pre-catalyst and K_2_CO_3_ as the base [18–19]. However, under these reaction conditions not any desired product was formed, and only the starting material BBRB was recovered after 20 h as a red solid, while that of the product should be yellow in color. Variations of reaction conditions (metal source, ligand, temp., etc.) were attempted but to no avail. The lack of reactivity is probably due to the strong negative inductive (−I) effect of the quaternary ammonium unit, which decreases the electron density on the phenoxide, making it only weakly nucleophilic (Scheme 2). Additionally, the zwitterionic structure (**I**) is in resonance with a neutral ketone structure (**II**), and natural resonance theory (NRT) analysis has suggested that the ketone structure is the predominant one [20].

To destroy such resonance, the isoquinoline core was thus reduced to tetrahydroisoqinoline **1** by NaBH_4_ [21]. To our delight, this reduced form of berberrubine smoothly underwent Ullmann-type cross coupling with iodobenzene, leading to the 9-O-Ph product **2a** in 65% yield under the aforementioned reaction conditions (Table 1).

**Table 1:** Optimization of the reaction conditions.^a^

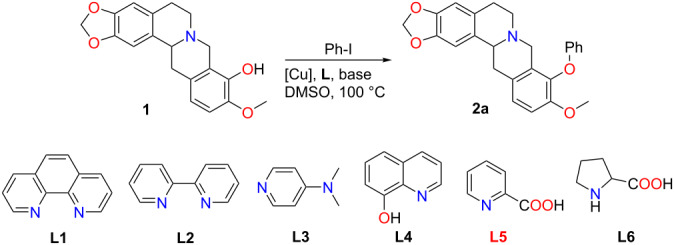						

Entry	[Cu]	Ligand	Solvent	Temp. (°C)	Time (h)	Yield (%)^b^

1	CuI	L1	DMSO	80	24	65
2	CuI	L2	DMSO	80	24	67
3	CuI	L3	DMSO	80	24	38
4	CuI	L4	DMSO	80	24	68
5	CuI	L5	DMSO	80	24	77
6	CuI	L6	DMSO	80	24	56
7	CuBr	L5	DMSO	80	24	74
8	CuCl	L5	DMSO	80	24	64
9	Cu(OAc)_2_	L5	DMSO	80	24	69
10	CuSO_4_	L5	DMSO	80	24	45
11	CuCl_2_	L5	DMSO	80	24	64
12	CuO	L5	DMSO	80	24	75
13	CuI	L5	DMF	80	24	68^c^
14	CuI	L5	DMA	80	24	63^c^
15	CuI	L5	dioxane	80	24	trace
16	CuI	L5	DMSO	60	24	49
**17**	**CuI**	**L5**	**DMSO**	**100**	**24**	**83 (72^c^)**
18	CuI	L5	DMSO	120	24	42
19	CuI	L5	DMSO	140	24	47
20	CuI	L5	DMSO	100	48	84

^a^Reaction conditions: **1** (0.10 mmol), PhI (0.20 mmol), [Cu] (0.01 mmol), L (0.02 mmol), base (0.10 mmol), solvent (1 mL). ^b^NMR yields for an average of two runs using mesitylene as an internal standard. ^c^Isolated yields for an average of two runs.

To improve the catalytic efficiency, multiple ligands were tested for their activities, among which the *κ*^2^-N,O-chelating ligand picolinic acid (**L5**) was found as the best one, affording **2a** in an enhanced yield of 77% (Table 1, entry 5). Both Cu(I) and Cu(II) are able to promote the reaction, however, CuI afforded the best results (Table 1, entry 5 and entries 7–12).

Elevating the temperature to 100 °C leads to 83% product formation (Table 1, entry 17). However, when the reaction temperature was further increased, the analysis of the crude product mixture revealed a complex NMR spectrum, among which one set of the signals is assignable to the 9-O-Ph BBR (**3a**, vide infra). This suggests a ready oxidation of **2a** to BBR derivative **3a** probably involving the solvent DMSO under such conditions (Supporting Information File 1, Figure S1). However, changing the solvent to DMF, DMA or 1,4-dioxane did not show any improvement in the yields (Table 1, entries 13–15). Also longer reaction times (48 h) had no significant effect on the product yield (Table 1, entry 20).

**Scheme 3:**
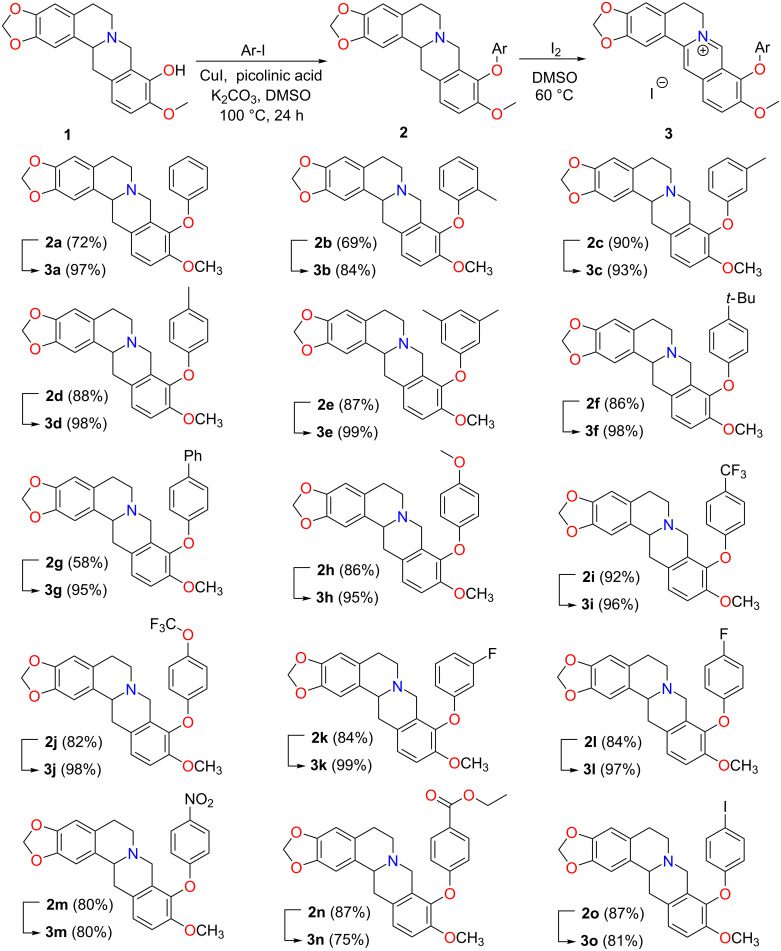
9-O-Aryl berberine scope via cross-coupling reaction.

Having the optimized reaction conditions in hand, the substrate scope was investigated using various aryl iodides (Scheme 3). Both, electron-rich and electron-deficient aryl iodides afforded the corresponding 9-O-arylated tetrahydroberberines **2a**–**o** in good to decent yields. Also, nitro and ester substituents were well-tolerated under these conditions giving the respective BBR derivatives in high yields. More important, the method allows the incorporation of pharmaceutically highly interesting fluorine-substituted aryl groups into the BBR skeleton.

**Scheme 4:**
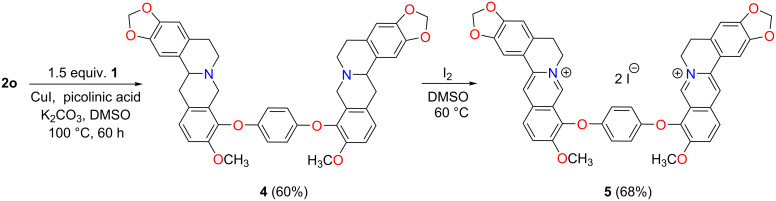
9-O-Ph-linked berberine dimer through double cross-coupling reaction.

Interestingly, only a single cross-coupling product was observed with 1,4-diiodobenzene, affording tetrahydroberberine analog **2o** in 87% yield. The doubly coupled product **4** was later accessed in 60% yield by repeating the reaction with **2o** as the starting material (Scheme 4).

The synthesized compounds **2a**–**o** and **4** are pale yellow solids with good solubility in most organic solvents such as diethyl ether, ethyl acetate, CH_2_Cl_2_, CHCl_3_, CH_3_CN and MeOH. Besides the appearance of the additional resonances from the aryl groups in the ^1^H NMR spectra, the disappearance of the singlet at 5.71 ppm characteristic for the O–H signal in **1** also confirmed their generation. The methylene protons are resonating as multiplets in the range of 4.00–2.00 ppm, and they are diastereotopic due to the restricted rotation of the hydroquinolizine rings. Both *anti*- and *syn*-rotamers were observed in a ratio of 3:1 in the ^1^H NMR spectrum of compound **4**.

As the quaternary ammonium unit is essential for biological activity, compounds **2a**–**o** and **4** were subsequently converted back to the respective 9-O-arylated berberines and 9-O-phenylene-bridged berberine dimer. Oxidation with DMSO*-d*_6_ was first attempted with **2a**. It showed good stability at ambient temperature and only 16% conversion to the 9-O-Ph berberine **3a** was noted after 72 h of heating at 100 °C (Supporting Information File 1, Figure S2). To promote this process I_2_ was used as the oxidant and provides the anion at the same time [22]. Following this procedure, the oxidation went to completion within 6 h as indicated by TLC (Scheme 3, Scheme 4), and no substitution on the aromatic backbone was observed as with Br_2_ [5]. This makes I_2_ a better oxidant for the oxidation of tetrahydroberberines.

Compounds **3a**–**o** are yellow solids, which are soluble in CH_2_Cl_2_, CH_3_CN, DMSO and MeOH. In contrast, the phenylene-bridged dimer **5** is only sparingly soluble in DMSO and MeOH. The formation of the products was firstly indicated by mass spectrometry (ESIMS), in which the respective [M − I]^+^ signal was observed as the base peak. The NMR spectra resembled those of BBR, with the methylene protons resonating as triplets in the range of 5.00–3.00 ppm. Only one rotamer was observed in the ^1^H NMR spectrum of **5**.

## Conclusion

We have presented the synthesis of unprecedented 9-(O)-aryl-substituted BBR through a copper-catalyzed cross coupling of tetrahydroberberrubine with aryl iodides and subsequent oxidation. The substrate scope is generally broad and various aryl groups have been introduced to the BBR structure. Considering the great abundance of aryl halides, the method allows the preparation of more diverse BBR derivatives in the future. The relevant biological study of the newly synthesized compounds is currently underway in our laboratory.

## Supporting Information

File 1 Experimental details and characterization data.
