# Supplementary material for: Synthesis of 9-O-arylated berberines via copper-catalyzed CAr–O coupling reactions
Source: Beilstein J Org Chem. 2019 Jul 15;15:1575–80. doi: 10.3762/bjoc.15.161 (PMC6664384; doi:10.3762/bjoc.15.161)
Supplement: File 1 — Experimental details and characterization data. [file Beilstein_J_Org_Chem-15-1575-s001.pdf]

BEILSTEIN JOURNAL OF ORGANIC CHEMISTRY

Supporting Information

for

Synthesis of 9-O-arylated berberines via copper-catalyzed C_{Ar}–O coupling reactions

Qiaoqiao Teng, Xinhui Zhu, Qianqian Guo, Weihua Jiang, Jiang Liu and Qi Meng

Beilstein J. Org. Chem. **2019,** *15,* 1575–1580. doi:10.3762/bjoc.15.161

Experimental details and characterization data

License and Terms: This is a supporting information file under the terms of the Creative Commons Attribution License (http://creativecommons.org/licenses/by/4.0). Please note that the reuse, redistribution and reproduction in particular requires that the authors and source are credited.
The license is subject to the *Beilstein Journal of Organic Chemistry* terms and conditions: (https://www.beilstein-journals.org/bjoc)

Table of Contents

Experimental section S1

NMR spectra S23

Figure S1: NMR analysis of reaction mixture at 140 °C S55

Figure S2: Oxidation of **2a** with DMSO S55

Selected examples of HRMS spectra S56

Representative IR spectra S58

References S60

General considerations

All operations were performed without taking precautions to exclude air or moisture unless otherwise stated. All solvents and chemicals were used as received. ^{1}H and ^{13}C NMR spectra were recorded at 298 K on a Bruker AVANCE spectrometer, and the chemical shifts (δ) were internally referenced to the residual solvent signals relative to tetramethylsilane. FTIR spectra were recorded on a Thermo Fischer Scientific Nicolet IS50 infrared spectrometer using KBr pellets. HRMS analyses were performed on an Agilent Technologies 6230 TOF spectrometer. Tetrahydroberberrubine (**1**) was prepared according to the literature procedure and its formation was confirmed by ^{1}H NMR spectroscopy.[1]

General procedure for the cross-coupling

A Schlenk tube was charged with a mixture of tetrahydroberberrubine (**1**, 228 mg, 0.70 mmol), potassium carbonate (97 mg, 0.70 mmol), copper(I) iodide (13 mg, 0.07 mmol), picolinic acid (17 mg, 0.14 mmol), aryl iodide (1.40 mmol, 2 equiv) and DMSO (6 mL) before it was evacuated and backfilled with N_2 for three times. The reaction mixture was stirred for 24 h at 100 °C and the resulting mixture was subjected to column chromatography (SiO_2, CH_2Cl_2/MeOH 200:1–150:1) to give the respective product **2a–o** as pale-yellow solids and **4** as yellow solid. Characterization data and the original spectra are presented below.

General procedure for the oxidation

A reaction tube was charged with **2** or **4** (0.20 mmol) and I_2 (152 mg, 0.6 mmol, double amount for **4**) in DMSO (2 mL). The reaction mixture was stirred at 60 °C until

consumption of **2**,**4** (TLC). Then, the solvent was removed by vacuum distillation before the residue was dissolved in CH_2Cl_2, extracted three times with saturated $NaHSO_3$, and twice with H_2O. The organic phase was dried over anhydrous Na_2SO_4, filtered, and dried in vacuo to give the respective products **3a–o** as yellow solids. As for **5**, it was purified by direct H_2O wash owing to its low solubility. Characterization data and the original spectra are presented below.

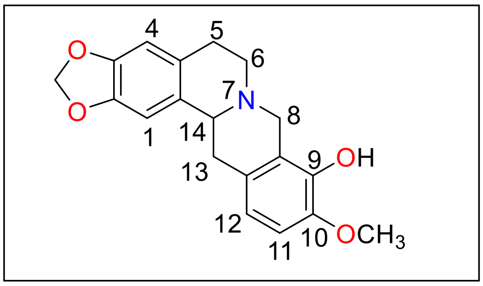

1, off-white solid, yield 62%. ^1H NMR (300 MHz, $CDCl_3$): δ 6.74 (s, 1 H, H-1), 6.73 (d, J=7.1 Hz, 1 H, H-11), 6.66 (d, J=7.1 Hz, 1 H, H-12), 6.59 (s, 1 H, H-4), 5.92 (s, 2 H, OCH_2O), 5.71 (s, 1 H, OH), 4.23 (d, 1 H, J=15.6 Hz, H-14), 3.86 (s, 3 H, OCH_3), 3.57-3.48 (m, 2 H, H-8), 3.25-3.06 (m, 3 H, H-5 & H-6 & H-13), 2.86-2.77 (m, 1 H, H-5), 2.67-2.60 (m, 2 H, H-6 & H-13).

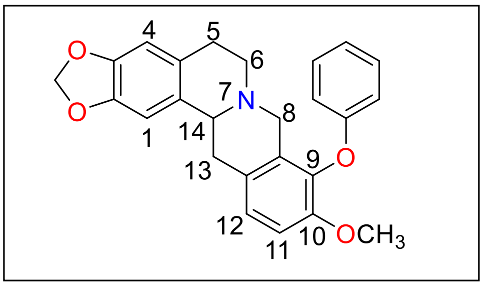

2a, pale yellow solid, m.p.: 144-146 °C, yield 72%. ^1H NMR (300 MHz, $CDCl_3$): δ 7.28-7.23 (m, 2 H, Ar-H), 7.04-6.96 (m, 2 H, Ar-H), 6.88-6.84 (m, 3 H, Ar-H), 6.74 (s, 1 H, H-1), 6.57 (s, 1 H, H-4), 5.91 (q, 2 H, J=1.4 Hz, OCH_2O), 4.07 (d, 1 H, J=15.9 Hz, H-8), 3.74 (s, 3 H, OCH_3), 3.56-3.51 (m, 1 H, H-14), 3.43 (d, 1 H, J=15.9 Hz, H-8), 3.31-3.24 (m, 1 H, H-13), 3.11-2.98 (m, 2 H, H-6 & H-5), 2.91-2.82 (m, 1 H, H-5), 2.63-2.48 (m, 2 H, H-

6 & H-13); $^{13}C\{^1H\}$ NMR (75 MHz, $CDCl_3$): δ 158.1, 150.4, 146.3, 146.1, 139.2, 130.8, 129.7, 129.6, 128.2, 127.9, 125.8, 121.7, 114.8, 111.2, 108.5, 105.6 (Ar-C), 100.9 (OCH_2O), 59.7 (C-14), 56.2 (OCH_3), 53.8 (C-8), 51.3 (C-6), 36.5 (C-13), 29.6 (C-5); HRMS (ESI): *m/z* for $C_{25}H_{24}NO_4^+$ $[M + H]^+$ calcd. 402.1705, found 402.1709.

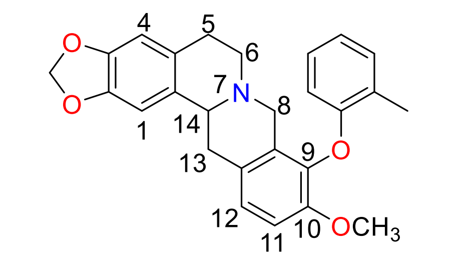

2b, pale yellow solid, m.p. 153-155 °C, yield 69%. ^1H NMR (300 MHz, $CDCl_3$): δ 7.20 (dd, 1 H, *J*=0.8 Hz, *J*=7.3 Hz, Ar-H), 7.03-6.96 (m, 2 H, Ar-H), 6.91-6.85 (m, 2 H, Ar-H), 6.74 (s, 1 H, H-1), 6.57 (s, 1 H, H-4), 6.44-6.41 (m, 1 H, Ar-H), 5.92 (q, 2 H, *J*=1.4 Hz, OCH_2O), 4.05 (d, 1 H, *J*=15.8 Hz, H-8), 3.72 (s, 3 H, OCH_3), 3.56-3.52 (m, 1 H, H-14), 3.43 (d, 1 H, *J*=15.8 Hz, H-8), 3.30-3.24 (m, 1 H, H-13), 3.12-3.03 (m, 2 H, H-6 & H-5), 2.91-2.82 (m, 1 H, H-5), 2.64-2.48 (m, 2 H, H-6 & H-13), 2.44 (s, 3 H, CH_3); $^{13}C\{^1H\}$ NMR (75 MHz, $CDCl_3$): δ 156.1, 150.5, 146.3, 146.1, 139.9, 131.0, 130.8, 129.4, 128.2, 127.9, 126.8, 126.3, 125.6, 121.4, 112.4, 111.4, 108.5, 105.6 (Ar-C), 100.9 (OCH_2O), 59.7 (C-14), 56.3 (OCH_3), 53.7 (C-8), 51.3 (C-6), 36.5 (C-13), 29.6 (C-5), 16.4 (CH_3); HRMS (ESI): *m/z* for $C_{26}H_{26}NO_4^+$ $[M + H]^+$ calcd. 416.1862, found 416.1861.

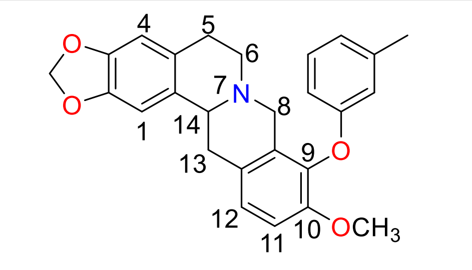

2c, pale yellow solid, m.p. 158-160 °C, yield 90%. ^1H NMR (300 MHz, $CDCl_3$): δ 7.12 (t, 1 H, *J*=7.8 Hz, Ar-H), 7.02 (d, 1 H, *J*=8.4 Hz, H-11), 6.87 (d, 1 H, *J*=8.4 Hz, H-12), 6.80 (d, 1

H, J=7.5 Hz, Ar-H), 6.73 (s, 1 H, H-1), 6.70-6.69 (m, 1 H, Ar-H), 6.62 (dd, 1 H, J=2.4 Hz, J=8.2 Hz, Ar-H), 6.57 (s, 1 H, H-4), 5.92 (q, 2 H, J=1.4 Hz, OCH_2O), 4.06 (d, 1 H, J=15.9 Hz, H-8), 3.74 (s, 3 H, OCH_3), 3.56-3.51 (m, 1 H, H-14), 3.43 (d, 1 H, J=15.9 Hz, H-8), 3.30-3.24 (m, 1 H, H-13), 3.11-2.99 (m, 2 H, H-6 & H-5), 2.91-2.81 (m, 1 H, H-5), 2.62-2.49 (m, 2 H, H-6 & H-13), 2.30 (s, 3 H, CH_3); $^{13}C\{^1H\}$ NMR (75 MHz, $CDCl_3$): δ 158.1, 150.5, 146.3, 146.1, 139.7, 139.4, 130.8, 129.8, 129.3, 128.2, 127.9, 125.7, 122.6, 115.5, 111.7, 111.3, 108.6, 105.6 (Ar-C), 100.9 (OCH_2O), 59.7 (C-14), 56.3 (OCH_3), 53.8 (C-8), 51.4 (C-6), 36.6 (C-13), 29.6 (C-5), 21.6 (CH_3); HRMS (ESI): m/z for $C_{26}H_{26}NO_4^+$ $[M + H]^+$ calcd. 416.1862, found 416.1837.

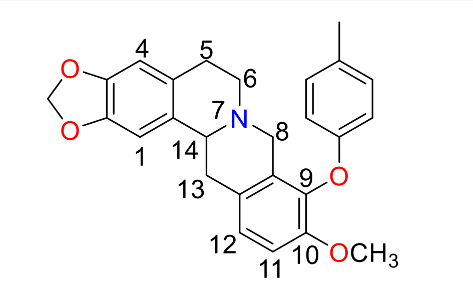

2d, pale yellow solid, m.p. 142-144 °C, yield 88%. ^1H NMR (300 MHz, $CDCl_3$): δ 7.06-7.00 (m, 3 H, H-11 & Ar-H), 6.86 (d, 1 H, J=8.4, H-12), 6.75-6.73 (m, 3 H, H-1 & Ar-H), 6.57 (s, 1 H, H-4), 5.91 (q, 2 H, J=1.4 Hz, OCH_2O), 4.07 (d, 1 H, J=15.9 Hz, H-8), 3.73 (s, 3 H, OCH_3), 3.55-3.50 (m, 1 H, H-14), 3.42 (d, 1 H, J=15.9 Hz, H-8), 3.30-3.23 (m, 1 H, H-13), 3.12-3.02 (m, 2 H, H-6 & H-5), 2.90-2.81 (m, 1 H, H-5), 2.63-2.48 (m, 2 H, H-13 & H-6), 2.28 (s, 3 H, CH_3); $^{13}C\{^1H\}$ NMR (75 MHz, $CDCl_3$): δ 156.0, 150.5, 146.3, 146.1, 139.5, 130.9, 130.8, 130.1, 129.8, 128.2, 127.9, 125.7, 114.5, 111.3, 108.6, 105.6 (Ar-C), 100.9 (OCH_2O), 59.7 (C-14), 56.3 (OCH_3), 53.8 (C-8), 51.4 (C-6), 36.5 (C-13), 29.6 (C-5), 20.7 (CH_3); HRMS (ESI): m/z for $C_{26}H_{26}NO_4^+$ $[M +H]^+$ calcd. 416.1862, found 416.1859.

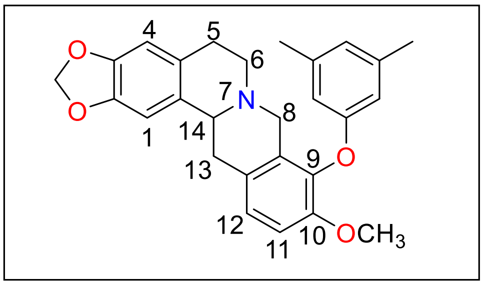

2e, pale yellow solid, m.p. 193-195 °C, yield 87%. ^{1}H NMR (300 MHz, $CDCl_3$): δ 7.02 (d, 1 H, J=8.4 Hz, H-11), 6.87 (d, 1 H, J=8.4 Hz, H-12), 6.74 (s, 1 H, H-1), 6.63 (s, 1 H, Ar-H), 6.57 (s, 1 H, H-4), 6.47 (s, 2 H, Ar-H), 5.92 (q, 2 H, J=1.4 Hz, OCH_2O), 4.07 (d, 1 H, J=15.9 Hz, H-8), 3.74 (s, 3 H, OCH_3), 3.57-3.52 (m, 1 H, H-14), 3.45 (d, 1 H, J=15.9 Hz, H-8), 3.31-3.25 (m, 1 H, H-13), 3.12-2.98 (m, 2 H, H-6 & H-5), 2.92-2.83 (m, 1 H, H-5), 2.64-2.49 (m, 2 H, H-13 & H-6), 2.25 (s, 6 H, CH_3); $^{13}C\{^{1}H\}$ NMR (75 MHz, $CDCl_3$): δ 158.1, 150.5, 146.3, 146.1, 139.4, 139.3, 130.8, 129.7, 128.1, 127.9, 125.6, 123.6, 112.4, 111.3, 108.5, 105.6 (Ar-C), 100.9 (OCH_2O), 59.6 (C-14), 56.3 (OCH_3), 53.8 (C-8), 51.3 (C-6), 36.5 (C-13), 29.5 (C-5), 21.5 (CH_3); HRMS (ESI): m/z for $C_{27}H_{28}NO_4^+$ $[M + H]^+$ calcd. 430.2018, found 430.2014.

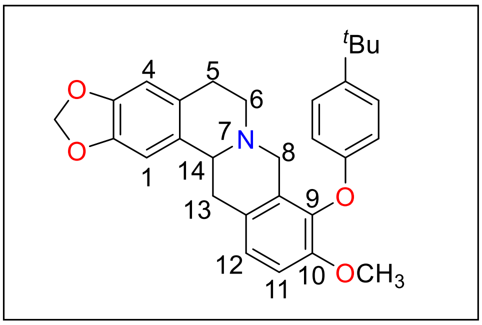

2f, pale yellow solid, m.p. 144-146 °C, yield 86%. ^{1}H NMR (300 MHz, $CDCl_3$): δ 7.24-7.22 (m, 2 H, Ar-H), 7.01 (d, 1 H, J=8.4 Hz, H-11), 6.86 (d, 1 H, J=8.4 Hz, H-12), 6.79-6.75 (m, 2 H, Ar-H), 6.73 (s, 1 H, H-1), 6.57 (s, 1 H, H-4), 5.91 (q, 2 H, J=1.4 Hz, OCH_2O), 4.07 (d, 1 H, J=15.9 Hz, H-8), 3.74 (s, 3 H, OCH_3), 3.56-3.51 (m, 1 H, H-14), 3.43 (d, 1 H, J=15.9 Hz, H-8), 3.30-3.23 (m, 1 H, H-13), 3.11-3.03 (m, 2 H, H-6 & H-5), 2.90-2.81 (m, 1 H, H-5), 2.63-2.47 (m, 2 H, H-13 & H-6), 1.29 (s, 9 H, CH_3); $^{13}C\{^{1}H\}$ NMR (75 MHz, $CDCl_3$): δ 155.8, 150.5, 146.3, 146.1,

144.3, 139.6, 130.8, 129.7, 128.2, 127.9, 126.4, 125.7, 114.1, 111.3, 108.5, 105.6 (Ar-C), 100.9 (OCH_2O), 59.7 (C-14), 56.3 (OCH_3), 53.8 (C-8), 51.3 (C-6), 36.5 (C-13), 34.2 (*C*(CH_3)$_3$), 31.7 (CH_3), 29.6 (C-5); HRMS (ESI): *m/z* for $C_{29}H_{32}NO_4^+$ $[M + H]^+$ calcd. 458.2331, found 458.2333.

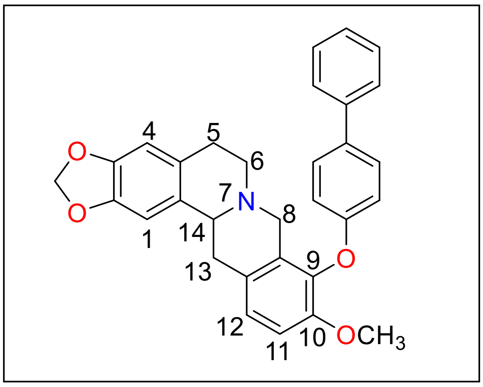

2g, pale yellow solid, m.p. 169-171 °C, yield 58%. ^1H NMR (300 MHz, $CDCl_3$): δ 7.56-7.53 (m, 2 H, Ar-H), 7.50-7.47 (m, 2 H, Ar-H), 7.40-7.38 (m, 2 H, Ar-H), 7.32-7.29 (m, 1 H, Ar-H), 7.05 (d, 1 H, *J*=8.4 Hz, H-11), 6.93-6.88 (m, 3 H, H-12 & Ar-H), 6.74 (s, 1 H, H-1), 6.57 (s, 1 H, H-4), 5.92 (q, 2 H, *J*=1.4 Hz, OCH_2O), 4.10 (d, 1 H, *J*=15.9 Hz, H-8), 3.76 (s, 3 H, OCH_3), 3.57-3.53 (m, 1 H, H-14), 3.47 (d, 1 H, *J*=15.9 Hz, H-8), 3.32-3.26 (m, 1 H, H-13), 3.12-3.00 (m, 2 H, H-6 & H-5), 2.92-2.83 (m, 1 H, H-5), 2.65-2.50 (m, 2 H, H-13 & H-6); $^{13}C\{^1H\}$ NMR (75 MHz, $CDCl_3$): δ 157.7, 150.4, 146.3, 146.1, 140.8, 139.2, 134.8, 130.8, 129.7, 128.8, 128.32, 128.26, 127.9, 126.89, 126.85, 125.9, 115.1, 111.3, 108.5, 105.6 (Ar-C), 100.9 (OCH_2O), 59.7 (C-14), 56.2 (OCH_3), 53.8 (C-8), 51.4 (C-6), 36.5 (C-13), 29.6 (C-5); HRMS (ESI): *m/z* for $C_{31}H_{28}NO_4^+$ $[M + H]^+$ calcd. 478.2018, found 478.2015.

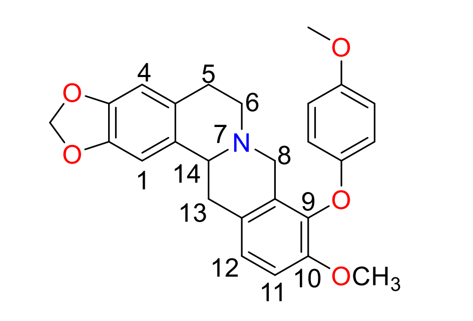

2h, pale yellow solid, m.p. 136-138 °C, yield 86%. ^{1}H NMR (300 MHz, $CDCl_3$): δ 7.01 (d, 1 H, J=8.4 Hz, H-11), 6.86 (d, 1 H, J=8.4 Hz, H-12), 6.78 (s, 4 H, Ar-H), 6.73 (s, 1 H, H-1), 6.57 (s, 1 H, H-4), 5.91 (q, 2 H, J=1.4 Hz, OCH_2O), 4.08 (d, 1 H, J=15.9 Hz, H-8), 3.76 (s, 3 H, OCH_3), 3.74 (s, 3 H, OCH_3), 3.56-3.51 (m, 1 H, H-14), 3.42 (d, 1 H, J=15.9 Hz, H-8), 3.30-3.23 (m, 1 H, H-13), 3.12-2.98 (m, 2 H, H-6 & H-5), 2.90-2.81 (m, 1 H, H-5), 2.63-2.48 (m, 2 H, H-13 & H-6); $^{13}C\{^{1}H\}$ NMR (75 MHz, $CDCl_3$): δ 154.4, 152.2, 150.5, 146.3, 146.1, 139.9, 130.8, 129.7, 128.2, 127.9, 125.6, 115.5, 114.7, 111.4, 108.6, 105.6 (Ar-C), 100.9 (OCH_2O), 59.7 (C-14), 56.3 (OCH_3), 55.8 (OCH_3), 53.9 (C-8), 51.3 (C-6), 36.5 (C-13), 29.6 (C-5); HRMS (ESI): m/z for $C_{26}H_{26}NO_5^{+}$ $[M + H]^{+}$ calcd. 432.1811, found 432.1812.

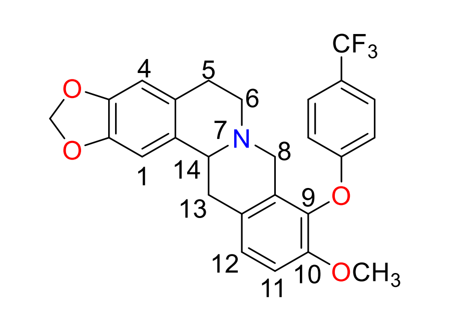

2i, pale yellow solid, m.p. 161-163 °C, yield 92%. ^{1}H NMR (300 MHz, $CDCl_3$): δ 7.51 (d, 2 H, J=8.5 Hz, Ar-H), 7.06 (d, 1 H, J=8.4 Hz, H-11), 6.93-6.87 (m, 3 H, H-12 & Ar-H), 6.73 (s, 1 H, H-1), 6.57 (s, 1 H, H-4), 5.91 (q, 2 H, J=1.4 Hz, OCH_2O), 4.02 (d, 1 H, J=15.9 Hz, H-8), 3.73 (s, 3 H, OCH_3), 3.56-3.51 (m, 1 H, H-14), 3.42 (d, 1 H, J=15.9 Hz, H-8), 3.31-3.24 (m, 1 H, H-13), 3.12-3.02 (m, 2 H, H-6 & H-5), 2.90-2.82 (m, 1 H, H-5), 2.64-2.50 (m, 2 H, H-13 & H-6); $^{13}C\{^{1}H\}$ NMR (75 MHz, $CDCl_3$): δ 160.5, 150.0, 146.3, 146.1, 138.5, 130.6, 129.4, 128.4, 127.8,

127.1 (q, *J*=3.7 Hz), 126.4, 124.5 (q, *J*=269.6 Hz), 124.0 (q, *J*=32.5 Hz), 114.9, 111.2, 108.5, 105.6 (Ar-C & CF_3), 100.9 (OCH_2O), 59.6 (C-14), 56.1 (OCH_3), 53.7 (C-8), 51.3 (C-6), 36.5 (C-13), 29.5 (C-5); ^{19}F NMR (282 MHz, $CDCl_3$): δ -61.5; HRMS (ESI): *m/z* for $C_{26}H_{23}F_3NO_4^+$ $[M + H]^+$ calcd. 470.1579, found 470.1592.

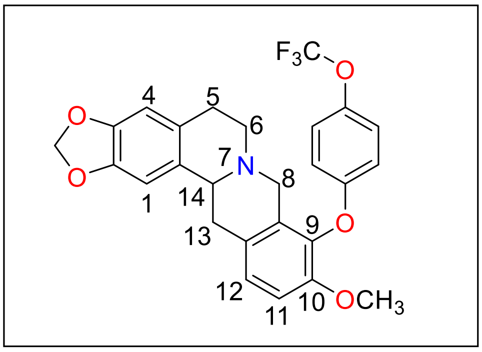

2j, pale yellow solid, m.p. 119-121 °C, yield 82%. ^1H NMR (300 MHz, $CDCl_3$): δ 7.11-7.02 (m, 3 H, H-11 & Ar-H), 6.88-6.82 (m, 3 H, H-12 & Ar-H), 6.73 (s, 1 H, H-1), 6.57 (s, 1 H, H-4), 5.91 (q, 2 H, *J*=1.4 Hz, OCH_2O), 4.03 (d, 1 H, *J*=15.9 Hz, H-8), 3.74 (s, 3 H, OCH_3), 3.56-3.51 (m, 1 H, H-14), 3.43 (d, 1 H, *J*=15.9 Hz, H-8), 3.30-3.24 (m, 1 H, H-13), 3.10-2.99 (m, 2 H, H-6 & H-5), 2.90-2.81 (m, 1 H, H-5), 2.64-2.49 (m, 2 H, H-13 & H-6); $^{13}C\{^1H\}$ NMR (75 MHz, $CDCl_3$): δ 156.4, 150.1, 146.3, 146.1, 143.5 (br-s, CF_3-O-*C*), 139.0, 130.6, 129.4, 128.3, 127.8, 126.1, 122.5 (br-s, CF_3-O-C-*C*H), 115.6, 111.2, 108.5, 105.5 (Ar-C, the CF_3 quartet was not resolved), 100.9 (OCH_2O), 59.6 (C-14), 56.0 (OCH_3), 53.7 (C-8), 51.3 (C-6), 36.4 (C-13), 29.5 (C-5); ^{19}F NMR (282 MHz, $CDCl_3$): δ -58.2; HRMS (ESI): *m/z* for $C_{26}H_{23}F_3NO_5^+$ $[M + H]^+$ calcd. 486.1528, found 486.1531.

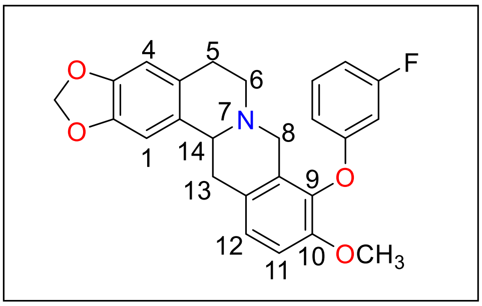

2k, pale yellow solid, m.p. 106-108 °C, yield 84%. ^{1}H NMR (300 MHz, $CDCl_3$): *δ* 7.23-7.16 (m, 1 H, Ar-H), 7.04 (d, 1 H, *J*=8.4 Hz, H-11), 6.87 (d, 1 H, *J*=8.4 Hz, H-12), 6.73-6.64 (m, 3 H, H-1 & Ar-H), 6.58-6.53 (m, 2 H, H-4 & Ar-H), 5.92 (q, 2 H, *J*=1.4 Hz, OCH_2O), 4.04 (d, 1 H, *J*=15.9 Hz, H-8), 3.75 (s, 3 H, OCH_3), 3.56-3.51 (m, 1 H, H-14), 3.42 (d, 1 H, *J*=15.9 Hz, H-8), 3.30-3.24 (m, 1 H, H-13), 3.12-2.99 (m, 2 H, H-6 & H-5), 2.90-2.81 (m, 1 H, H-5), 2.64-2.48 (m, 2 H, H-13 & H-6); $^{13}C\{^{1}H\}$ NMR (75 MHz, $CDCl_3$): *δ* 163.7 (d, *J*=243.9 Hz), 159.3 (d, *J*=10.7 Hz), 150.1, 146.3, 146.1, 138.8, 130.6, 130.3 (d, *J*=9.8 Hz), 129.5, 128.3, 127.8, 126.1, 111.2, 110.6 (d, *J*=2.8 Hz), 108.6 (d, *J*=21.2 Hz), 108.5, 105.6, 102.7 (d, *J*=25.2 Hz) (Ar-C), 100.9 (OCH_2O), 59.6 (C-14), 56.1 (OCH_3), 53.7 (C-8), 51.3 (C-6), 36.4 (C-13), 29.5 (C-5); ^{19}F NMR (282 MHz, $CDCl_3$): *δ* -111.7; HRMS (ESI): *m/z* for $C_{25}H_{23}FNO_4^+$ $[M + H]^+$ calcd. 420.1611, found 420.1608.

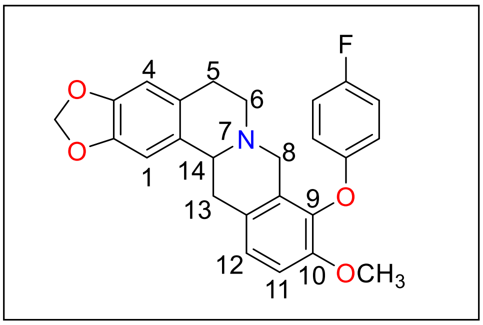

2l, pale yellow solid, m.p. 129-131 °C, yield 84%. ^{1}H NMR (300 MHz, $CDCl_3$): *δ* 7.02 (d, 1 H, *J*=8.4 Hz, H-11), 6.96-6.90 (m, 2 H, Ar-H), 6.86 (d, 1 H, *J*=8.4 Hz, H-12), 6.81 (m, 2 H, Ar-H), 6.73 (s, 1 H, H-1), 6.57 (s, 1 H, H-4), 5.92 (q, 2 H, *J*=1.4 Hz, OCH_2O), 4.05 (d, 1 H, *J*=15.9 Hz, H-8), 3.73 (s, 3 H, OCH_3), 3.56-3.51 (m, 1 H, H-14), 3.42 (d, 1 H, *J*=15.9 Hz, H-8), 3.30-3.23 (m, 1 H, H-13),

3.12-2.98 (m, 2 H, H-6 & H-5), 2.90-2.81 (m, 1 H, H-6), 2.64-2.48 (m, 2 H, H-13 & H-6); $^{13}C\{^1H\}$ NMR (75 MHz, $CDCl_3$): δ 157.8 (d, J=237.3 Hz), 154.1 (d, J=1.8 Hz), 150.2, 146.3, 146.1, 139.4, 130.6, 129.5, 128.3, 127.8, 125.9, 115.9 (d, J=31.3 Hz), 115.7 (d, J=8.0 Hz), 111.3, 108.5, 105.6 (Ar-C), 100.9 (OCH_2O), 59.6 (C-14), 56.1 (OCH_3), 53.8 (C-8), 51.3 (C-6), 36.4 (C-13), 29.5 (C-5); ^{19}F NMR (282 MHz, $CDCl_3$): δ -123.1; HRMS (ESI): m/z for $C_{25}H_{23}FNO_4^+$ $[M + H]^+$ calcd. 420.1611, found 420.1615.

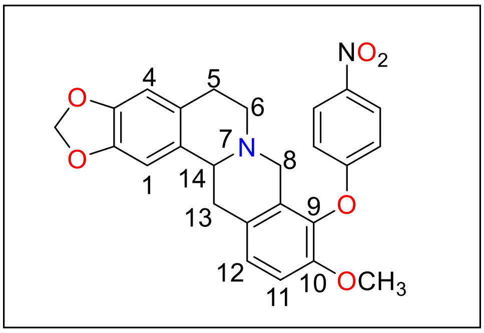

2m, brownish yellow solid, m.p. 182-184 °C, yield 80%. ^1H NMR (300 MHz, $CDCl_3$): δ 8.20-8.14 (m, 2 H, Ar-H), 7.08 (d, 1 H, J=8.4 Hz, H-11), 6.94-6.88 (m, 3 H, H-12 & Ar-H), 6.73 (s, 1 H, H-1), 6.57 (s, 1 H, H-4), 5.92 (q, 2 H, J=1.4 Hz, OCH_2O), 3.99 (d, 1 H, J=15.8 Hz, H-8), 3.73 (s, 3 H, OCH_3), 3.57-3.52 (m, 1 H, H-14), 3.43 (d, 1 H, J=15.8 Hz, H-8), 3.32-3.25 (m, 1 H, H-13), 3.12-2.98 (m, 2 H, H-6 & H-5), 2.91-2.82 (m, 1 H, H-5), 2.64-2.50 (m, 2 H, H-13 & H-6); $^{13}C\{^1H\}$ NMR (75 MHz, $CDCl_3$): δ 163.0, 149.7, 146.4, 146.2, 142.5, 138.2, 130.5, 129.1, 128.5, 127.8, 126.8, 126.1, 115.1, 111.2, 108.6, 105.6 (Ar-C), 101.0 (OCH_2O), 59.6 (C-14), 56.1 (OCH_3), 53.6 (C-8), 51.4 (C-6), 36.4 (C-13), 29.5 (C-5); HRMS (ESI): m/z for $C_{25}H_{23}N_2O_6^+$ $[M + H]^+$ calcd. 447.1556, found 447.1553.

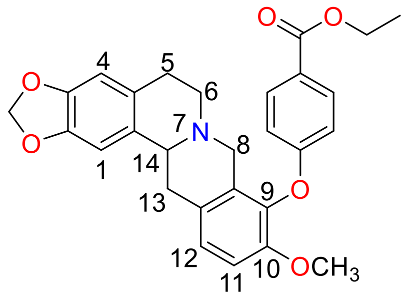

2n, pale yellow solid, m.p. 124-126 °C, yield 87%. ^{1}H NMR (300 MHz, $CDCl_3$): δ 7.99-7.94 (m, 2 H, Ar-H), 7.05 (d, 1 H, J=8.4 Hz, H-11), 6.89-6.86 (m, 3 H, H-12 & Ar-H), 6.73 (s, 1 H, H-1), 6.56 (s, 1 H, H-4), 5.91 (q, 2 H, J=1.4 Hz, OCH_2O), 4.33 (q, 2 H, J=7.1 Hz, CH_2), 4.02 (d, 1 H, J=15.9 Hz, H-8), 3.72 (s, 3 H, OCH_3), 3.55-3.50 (m, 1 H, H-14), 3.41 (d, 1 H, J=15.9 Hz, H-8), 3.30-3.24 (m, 1 H, H-13), 3.11-2.98 (m, 2 H, H-6 & H-5), 2.90-2.81 (m, 1 H, H-5), 2.63-2.48 (m, 2 H, H-13 & H-6), 1.36 (t, 3 H, J=7.1 Hz, CH_3); $^{13}C\{^{1}H\}$ NMR (75 MHz, $CDCl_3$): δ 166.4 (CO), 161.7, 150.0, 146.3, 146.1, 138.6, 131.7, 130.6, 129.3, 128.3, 127.8, 126.2, 124.1, 114.6, 111.2, 108.5, 105.6 (Ar-C), 100.9 (OCH_2O), 60.8 (CH_2), 59.6 (C-14), 56.1 (OCH_3), 53.7 (C-8), 51.3 (C-6), 36.4 (C-13), 29.5 (C-5), 14.5 (CH_3); HRMS (ESI): m/z for $C_{28}H_{28}NO_6^+$ $[M + H]^+$ calcd. 474.1917, found 474.1919.

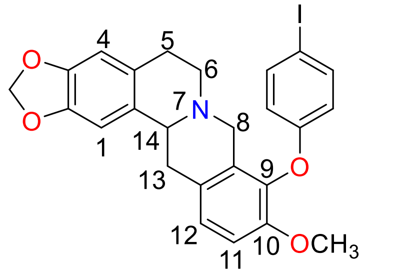

2o, pale yellow solid, m.p. 124-126 °C, yield 87%. ^{1}H NMR (500 MHz, $CDCl_3$): δ 7.53-7.50 (m, 2 H, Ar-H), 7.03 (d, 1 H, J=8.4 Hz, H-11), 6.86 (d, 1 H, J=8.4 Hz, H-12), 6.73 (s, 1 H, H-1), 6.64-6.61 (m, 2 H, Ar-H), 6.57 (s, 1 H, H-4), 5.92 (q, 2 H, J=1.4 Hz, OCH_2O), 4.02 (d, 1 H, J=15.9 Hz, H-8), 3.73 (s, 3 H, OCH_3), 3.55-3.52 (m, 1 H, H-14), 3.41 (d, 1 H, J=15.9 Hz, H-8), 3.29-3.25 (m, 1 H, H-13), 3.10-3.03 (m, 2 H, H-6 & H-5), 2.89-2.83 (m, 1 H, H-5), 2.63-2.51 (m, 2 H, H-13 & H-

6); $^{13}C\{^1H\}$ NMR (75 MHz, $CDCl_3$): δ 158.0, 150.1, 146.3, 146.1, 138.8, 138.4, 130.6, 129.4, 128.3, 127.9, 126.1, 117.2, 111.2, 108.6, 105.6 (Ar-C), 100.9 (OCH_2O), 84.0 (Ar-C), 59.6 (C-14), 56.2 (OCH_3), 53.7 (C-8), 51.3 (C-6), 36.4 (C-13), 29.5 (C-5); HRMS (ESI): *m/z* for $C_{28}H_{28}NO_6^+$ $[M + H]^+$ calcd. 528.0672, found 528.0683.

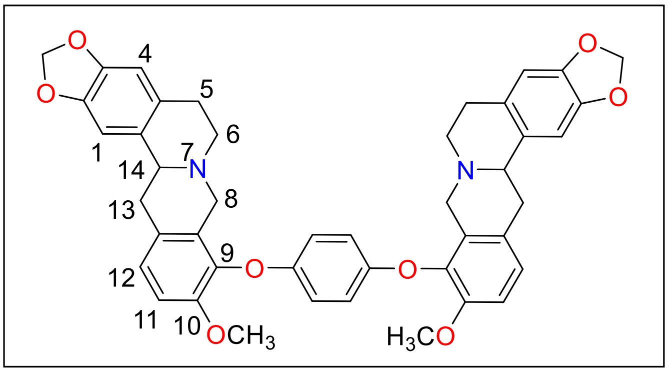

4, brownish yellow solid, m.p.: 293-295 °C, yield 60%. ^1H NMR (300 MHz, $CDCl_3$): δ 6.99 (d, 2 H, *J*=8.4 Hz, H-11), 6.88-6.77 (m, 3 H, H-12 & Ar-H), 6.73-6.72 (m, 5 H, Ar-H), 6.58 (d, 2 H, *J*=6.5 Hz, Ar-H), 5.91 (br-s, 4 H, OCH_2O), 4.24, 4.07 (m, 2 H, 1:3, H-8), 3.85, 3.74 (s, 6 H, 1:3, OCH_3), 3.55-3.51 (m, 2 H, H-14), 3.45-3.39 (m, 2 H, H-8), 3.28-3.17 (m, 2 H, H-13), 3.12-3.03 (m, 4 H, H-6 & H-5), 2.88-2.77 (m, 2 H, H-5), 2.68-2.49 (m, 4 H, H-13 & H-6); $^{13}C\{^1H\}$ NMR (75 MHz, $CDCl_3$): δ 152.73, 152.70, 150.5, 146.3, 146.1, 139.9, 139.8, 130.8, 129.6, 128.2, 127.9, 125.7, 115.6, 111.3, 108.5, 105.6 (Ar-C), 100.9 (OCH_2O), 59.6 (C-14), 56.2 (OCH_3), 53.9 (C-8), 51.4 (C-6), 36.5 (C-13), 29.6 (C-5); HRMS (ESI): *m/z* for $C_{44}H_{41}N_2O_8^+$ $[M + H]^+$ calcd. 725.2863, found 725.2861.

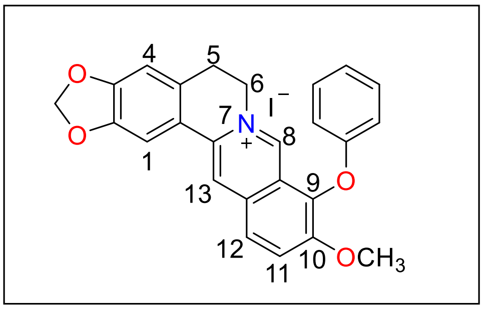

3a, yellow solid, m.p.: 242-244 °C, yield 97%. ^{1}H NMR (300 MHz, DMSO-d_6): δ 9.81 (s, 1 H, H-8), 9.06 (s, 1 H, H-13), 8.31 (d, 1 H, J=9.2 Hz, H-12), 8.20 (d, 1 H, J=9.2 Hz, H-11), 7.83 (s, 1 H, H-1), 7.38-7.33 (m, 2 H, Ar-H), 7.13-7.08 (m, 2 H, H-4 & Ar-H), 6.97-6.94 (m, 2 H, Ar-H), 6.18 (s, 2 H, OCH_2O), 4.91 (t, 2 H, J=6.0 Hz, H-6), 3.92 (s, 3 H, OCH_3), 3.18 (t, 2 H, J=6.0 Hz, H-5); ^{13}C{^{1}H} NMR (75 MHz, DMSO-d_6): δ 157.7, 150.8, 150.0, 147.7, 144.6, 138.1, 137.0, 133.4, 130.9, 129.7, 126.7, 125.8, 122.8, 122.0, 120.6, 120.4, 115.5, 108.4, 105.5 (Ar-C), 102.1 (OCH_2O), 57.0 (OCH_3), 55.3 (C-6), 26.2 (C-5); HRMS (ESI): m/z for $C_{25}H_{20}NO_4^+$ $[M - I]^+$ calcd. 398.1387, found 398.1392.

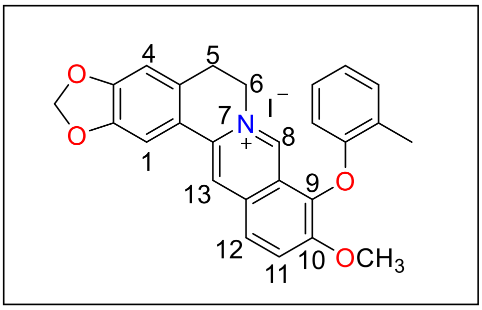

3b, yellow solid, m.p. 252-254 °C, yield 84%. ^{1}H NMR (300 MHz, DMSO-d_6): δ 9.78 (s, 1 H, H-8), 9.07 (s, 1 H, H-13), 8.30 (d, 1 H, J=9.2 Hz, H-12), 8.20 (d, 1 H, J=9.2 Hz, H-11), 7.82 (s, 1 H, H-1), 7.35-7.32 (m, 1 H, Ar-H), 7.08 (s, 1 H, H-4), 7.07-6.97 (m, 2 H, Ar-H), 6.44-6.41 (m, 1 H, Ar-H), 6.18 (s, 2 H, OCH_2O), 4.93 (t, 2 H, J=6.0 Hz, H-6), 3.89 (s, 3 H, OCH_3), 3.20 (t, 2 H, J=6.0 Hz, H-5), 2.50 (s, 3 H, CH_3); ^{13}C{^{1}H} NMR (75 MHz, DMSO-d_6): δ 156.0, 150.6, 149.9, 147.7, 144.6, 138.0, 137.6, 133.3, 131.1, 130.8, 126.9, 126.7, 126.3, 125.5, 122.6, 122.0, 120.6, 120.4, 113.2, 108.4, 105.5 (Ar-C), 102.1 (OCH_2O), 57.1 (OCH_3), 55.3 (C-6), 26.2 (C-5), 16.2 (CH_3); HRMS (ESI): m/z for $C_{26}H_{22}NO_4^+$ $[M - I]^+$ calcd. 412.1543, found 412.1550.

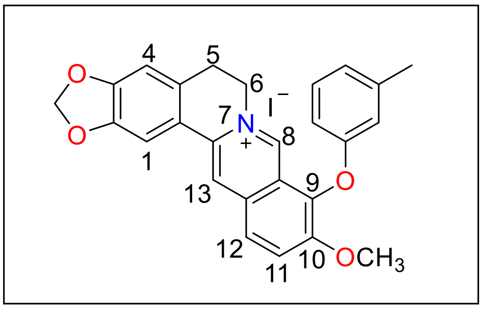

3c, yellow solid, m.p. 239-241 °C, yield 93%. ^{1}H NMR (300 MHz, DMSO-d_6): δ 9.77 (s, 1 H, H-8), 9.07 (s, 1 H, H-13), 8.31 (d, 1 H, *J*=9.2 Hz, H-12), 8.21 (d, 1 H, *J*=9.2 Hz, H-11), 7.82 (s, 1 H, H-1), 7.21 (t, 1 H, *J*=15.8 Hz, Ar-H), 7.08 (s, 1 H, H-4), 6.91 (d, 1 H, *J*=7.5 Hz, Ar-H), 6.80 (s, 1 H, Ar-H), 6.70 (dd, 1 H, *J*=2.5 Hz, *J*=8.2 Hz, Ar-H), 6.18 (s, 2 H, OCH_2O), 4.92 (t, 2 H, *J*=6.0 Hz, H-6), 3.92 (s, 3 H, OCH_3), 3.19 (t, 2 H, *J*=6.0 Hz, H-5), 2.27 (s, 3 H, CH_3); $^{13}C\{^{1}H\}$ NMR (75 MHz, DMSO-d_6): δ 157.7, 150.9, 149.9, 147.7, 144.6, 139.4, 138.1, 137.0, 133.4, 130.9, 129.4, 126.7, 125.8, 123.5, 122.0, 120.7, 120.4, 116.0, 112.4, 108.4, 105.5 (Ar-C), 102.2 (OCH_2O), 57.0 (OCH_3), 55.3 (C-6), 26.2 (C-5), 21.0 (CH_3); HRMS (ESI): *m/z* for $C_{26}H_{22}NO_4^+$ [M - I]$^+$ calcd. 412.1543, found 412.1548.

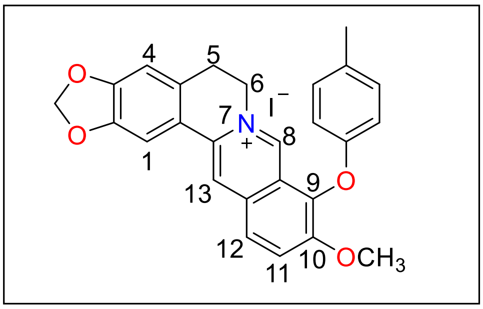

3d, yellow solid, m.p. 257-259 °C, yield 98%. ^{1}H NMR (500 MHz, DMSO-d_6): δ 9.77 (s, 1 H, H-8), 9.05 (s, 1 H, H-13), 8.30 (d, 1 H, *J*=9.2 Hz, H-12), 8.19 (d, 1 H, *J*=9.2 Hz, H-11), 7.83 (s, 1 H, H-1), 7.14 (d, 2 H, *J*=8.4 Hz, Ar-H), 7.08 (s, 1 H, H-4), 6.84 (d, 2 H, *J*=8.6 Hz, Ar-H), 6.18 (s, 2 H, OCH_2O), 4.91 (t, 2 H, *J*=6.0 Hz, H-6), 3.92 (s, 3 H, OCH_3), 3.18 (t, 2 H, *J*=6.0 Hz, H-5), 2.27 (s, 3 H, CH_3); $^{13}C\{^{1}H\}$ NMR (125 MHz, DMSO-d_6): δ 155.7, 150.9, 149.9, 147.7, 144.6, 138.1, 137.3, 133.4, 131.7, 130.9, 130.0, 126.7, 125.7, 122.0, 120.6, 120.4, 115.3, 108.4, 105.5 (Ar-

C), 102.1 (OCH_2O), 57.0 (OCH_3), 55.3 (C-6), 26.2 (C-5), 20.1 (CH_3); HRMS (ESI): m/z for $C_{26}H_{22}NO_4^+$ $[M - I]^+$ calcd. 412.1543, found 412.1550.

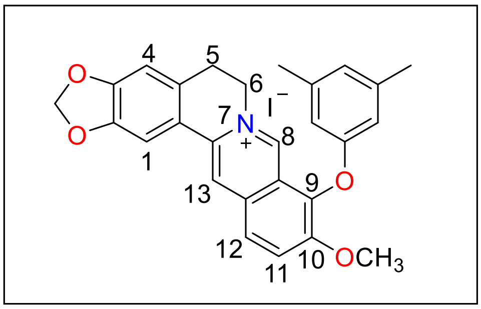

3e, yellow solid, m.p. 251-253 °C, yield 99%. ^{1}H NMR (300 MHz, DMSO-d_6): δ 9.74 (s, 1 H, H-8), 9.06 (s, 1 H, H-13), 8.31 (d, 1 H, J=9.2 Hz, H-12), 8.20 (d, 1 H, J=9.2 Hz, H-11), 7.82 (s, 1 H, H-1), 7.08 (s, 1 H, H-4), 6.73 (s, 1 H, Ar-H), 6.54 (s, 2 H, Ar-H), 6.18 (s, 2 H, OCH_2O), 4.91 (t, 2 H, J=6.0 Hz, H-6), 3.93 (s, 3 H, OCH_3), 3.18 (t, 2 H, J=6.0 Hz, H-5), 2.21 (s, 6 H, CH_3); ^{13}C{^{1}H} NMR (75 MHz, DMSO-d_6): δ 157.8, 150.9, 149.9, 147.7, 144.6, 139.0, 138.1, 137.0, 133.4, 130.9, 126.8, 125.7, 124.4, 122.1, 120.7, 120.4, 112.9, 108.4, 105.5 (Ar-C), 102.2 (OCH_2O), 57.1 (OCH_3), 55.3 (C-6), 26.2 (C-5), 20.9 (CH_3); HRMS (ESI): m/z for $C_{27}H_{24}NO_4^+$ $[M - I]^+$ calcd. 426.1700, found 426.1708.

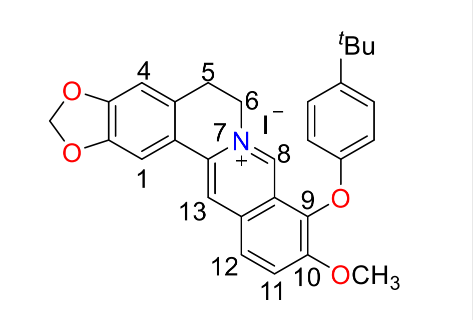

3f, yellow solid, m.p. 236-238 °C, yield 98%. ^{1}H NMR (300 MHz, DMSO-d_6): δ 9.77 (s, 1 H, H-8), 9.06 (s, 1 H, H-13), 8.31 (d, 1 H, J=9.2 Hz, H-12), 8.21 (d, 1 H, J=9.2 Hz, H-11), 7.82 (s, 1 H, H-1), 7.35 (d, 2 H, J=8.7 Hz, Ar-H), 7.07 (s, 1 H, H-4), 6.86 (d, 2 H, J=8.7 Hz, Ar-H), 6.18 (s, 2 H, OCH_2O), 4.91 (t, 2 H, J=6.0 Hz, H-6), 3.94 (s, 3 H, OCH_3), 3.18 (t, 2 H, J=6.0 Hz, H-5), 1.26 (s, 9 H, CH_3); ^{13}C{^{1}H} NMR (75 MHz, DMSO-d_6): δ 155.5, 151.0, 149.9, 147.7, 145.0, 144.6, 138.1,

137.0, 133.4, 130.9, 126.6, 126.4, 125.8, 122.1, 120.7, 120.4, 114.8, 108.4, 105.5 (Ar-C), 102.2 (OCH_2O), 57.1 (OCH_3), 55.3 (C-6), 34.0 (*C*($CH_3)_3$), 31.3 (CH_3), 26.2 (C-5); HRMS (ESI): *m/z* for $C_{29}H_{28}NO_4^+$ [M - I]$^+$ calcd. 454.2013, found 454.2020.

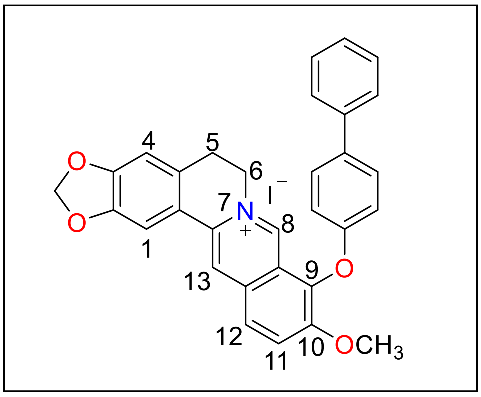

3g, yellow solid, m.p. 222-224 °C, yield 95%. ^{1}H NMR (300 MHz, DMSO-*d*$_6$): *δ* 9.83 (s, 1 H, H-8), 9.08 (s, 1 H, H-13), 8.34 (d, 1 H, *J*=9.2 Hz, H-12), 8.23 (d, 1 H, *J*=9.2 Hz, H-11), 7.83 (s, 1 H, H-1), 7.63 (t, 4 H, *J*=14.7 Hz, Ar-H), 7.45 (t, 2 H, *J*=7.5 Hz, Ar-H), 7.34 (t, 1 H, *J*=7.3 Hz, Ar-H), 7.05 (t, 3 H, *J*=8.7 Hz, H-4 & Ar-H), 6.18 (s, 2 H, OCH_2O), 4.93 (t, 2 H, *J*=6.0 Hz, H-6), 3.96 (s, 3 H, OCH_3), 3.19 (t, 2 H, *J*=6.0 Hz, H-5); ^{13}C{^{1}H} NMR (75 MHz, DMSO-*d*$_6$): *δ* 157.3, 150.9, 150.0, 147.7, 144.6, 139.5, 138.2, 136.8, 134.9, 133.4, 130.9, 129.0, 128.0, 127.1, 126.6, 126.4, 126.0, 122.0, 120.7, 120.4, 115.9, 108.4, 105.5 (Ar-C), 102.2 (OCH_2O), 57.1 (OCH_3), 55.3 (C-6), 26.2 (C-5); HRMS (ESI): *m/z* for $C_{31}H_{24}NO_4^+$ [M - I]$^+$ calcd. 474.1700, found 474.1708.

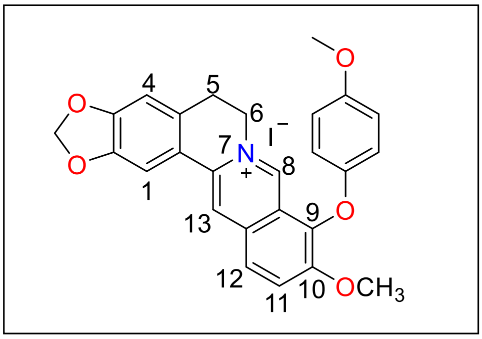

3h, yellow solid, m.p. 226-228 °C, yield 95%. ^{1}H NMR (300 MHz, DMSO-*d*$_6$): *δ* 9.80 (s, 1 H, H-8), 9.05 (s, 1 H, H-13), 8.30 (d, 1 H, *J*=9.2 Hz, H-12), 8.18 (d, 1 H, *J*=9.2 Hz, H-11), 7.82 (s, 1 H, H-1), 7.08 (s, 1 H, H-4), 6.90 (s, 4 H,

Ar-H), 6.18 (s, 2 H, OCH_2O), 4.92 (t, 2 H, *J*=6.0 Hz, H-6), 3.91 (s, 3 H, OCH_3), 3.72 (s, 3 H, OCH_3), 3.19 (t, 2 H, *J*=6.0 Hz, H-5); $^{13}C\{^1H\}$ NMR (75 MHz, DMSO-d_6): δ 154.9, 151.8, 150.9, 149.9, 147.7, 144.7, 138.1, 137.8, 133.3, 130.9, 126.8, 125.5, 122.1, 120.6, 120.4, 116.5, 114.6, 108.4, 105.5 (Ar-C), 102.2 (OCH_2O), 57.0 (OCH_3), 55.5 (OCH_3), 55.3 (C-6), 26.2 (C-5); HRMS (ESI): *m/z* for $C_{26}H_{22}NO_5^+$ $[M - I]^+$ calcd. 428.1492, found 428.1495.

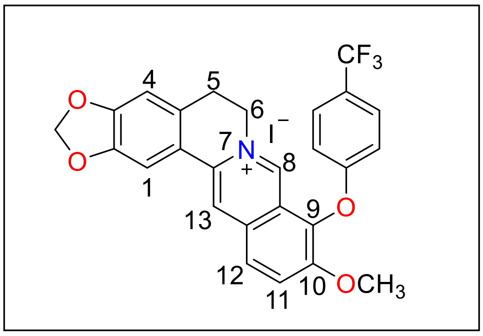

3i, yellow solid, m.p. 273-275 °C, yield 96%. ^1H NMR (300 MHz, DMSO-d$_6$): δ 9.83 (s, 1 H, H-8), 9.09 (s, 1 H, H-13), 8.35 (d, 1 H, *J*=9.2 Hz, H-12), 8.25 (d, 1 H, *J*=9.2 Hz, H-11), 7.82 (s, 1 H, H-1), 7.73 (d, 2 H, *J*=8.6 Hz, Ar-H), 7.16 (d, 2 H, *J*=8.46 Hz, Ar-H), 7.08 (s, 1 H, H-4), 6.18 (s, 2 H, OCH_2O), 4.91 (t, 2 H, *J*=6.0 Hz, H-6), 3.95 (s, 3 H, OCH_3), 3.19 (t, 2 H, *J*=6.0 Hz, H-5); $^{13}C\{^1H\}$ NMR (75 MHz, DMSO-d$_6$): δ 160.1, 150.7, 150.0, 147.7, 144.6, 138.2, 135.8, 133.4, 130.9, 127.2 (q, *J*=3.5 Hz), 126.5, 126.4, 124.3 (q, *J*=269.9 Hz), 123.4 (q, *J*=31.8 Hz), 121.6, 120.7, 120.3, 116.2, 108.4, 105.5 (Ar-C & CF_3), 102.2 (OCH_2O), 57.1 (OCH_3), 55.3 (C-6), 26.2 (C-5); ^{19}F NMR (282 MHz, $CDCl_3$): δ -60.1; HRMS (ESI): *m/z* for $C_{26}H_{19}F_3NO_4^+$ $[M - I]^+$ calcd. 466.1261, found 466.1273.

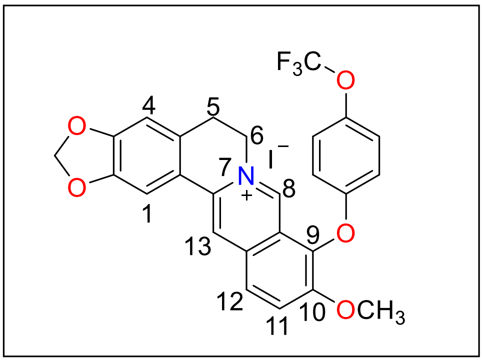

3j, yellow solid, m.p. 246-248 °C, yield 98%. ^{1}H NMR (300 MHz, DMSO-d_6): δ 9.84 (s, 1 H, H-8), 9.07 (s, 1 H, H-13), 8.33 (d, 1 H, J=9.2 Hz, H-12), 8.23 (d, 1 H, J=9.2 Hz, H-11), 7.83 (s, 1 H, H-1), 7.37 (d, 2 H, J=8.0 Hz, Ar-H), 7.07 (d, 3 H, J=8.0 Hz, Ar-H), 6.18 (s, 2 H, OCH_2O), 4.91 (t, 2 H, J=6.0 Hz, H-6), 3.94 (s, 3 H, OCH_3), 3.19 (t, 2 H, J=6.0 Hz, H-5); ^{13}C{^{1}H} NMR (75 MHz, DMSO-d_6): δ 156.2, 150.7, 150.0, 147.7, 144.6, 143.3 (d, J=1.4 Hz), 138.2, 136.5, 133.4, 130.8, 126.6, 126.2, 122.7 (br-s, CF_3-O-C-*C*H), 121.8, 120.6, 120.3, 120.1 (q, J=254.0 Hz), 117.0, 108.4, 105.5 (Ar-C & CF_3), 102.2 (OCH_2O), 57.1 (OCH_3), 55.3 (C-6), 26.2 (C-5); ^{19}F NMR (282 MHz, $CDCl_3$): δ -57.2; HRMS (ESI): m/z for $C_{26}H_{19}F_3NO_5^+$ [M - I]$^+$ calcd. 482.1210, found 482.1224.

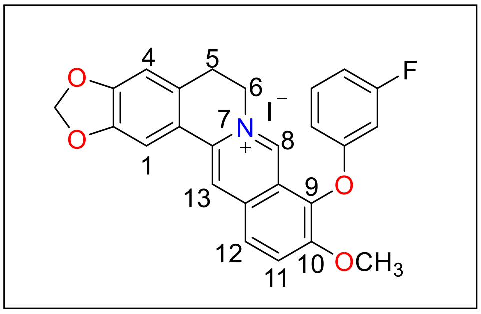

3k, yellow solid, m.p. 221-223 °C, yield 99%. ^{1}H NMR (300 MHz, DMSO-d_6): δ 9.81 (s, 1 H, H-8), 9.07 (s, 1 H, H-13), 8.32 (d, 1 H, J=9.2 Hz, H-12), 8.23 (d, 1 H, J=9.2 Hz, H-11), 7.83 (s, 1 H, H-1), 7.43-7.35 (m, 1 H, Ar-H), 7.09 (s, 1 H, H-4), 7.00-6.92 (m, 1 H, Ar-H), 6.87-6.80 (m, 2 H, Ar-H), 6.18 (s, 2 H, OCH_2O), 4.91 (t, 2 H, J=6.0 Hz, H-6), 3.95 (s, 3 H, OCH_3), 3.19 (t, 2 H, J=6.0 Hz, H-5); ^{13}C{^{1}H} NMR (75 MHz, DMSO-d_6): δ 162.8 (d, J=242.7 Hz), 158.8 (d, J=10.8 Hz), 150.7, 150.0, 147.7, 144.4, 138.1, 136.3, 133.4, 131.0 (d, J=9.9 Hz), 130.8, 126.6, 126.2, 121.8, 120.6, 120.3, 111.8 (d, J=2.1 Hz), 109.7

(d, J=21.2 Hz), 108.4, 105.5, 103.6 (d, J=25.4 Hz) (Ar-C), 102.2 (OCH_2O), 57.1 (OCH_3), 55.3 (C-6), 26.2 (C-5); ^{19}F NMR (282 MHz, $CDCl_3$): δ -111.4; HRMS (ESI): m/z for $C_{25}H_{19}FNO_4^+$ $[M - I]^+$ calcd. 416.1293, found 416.1296.

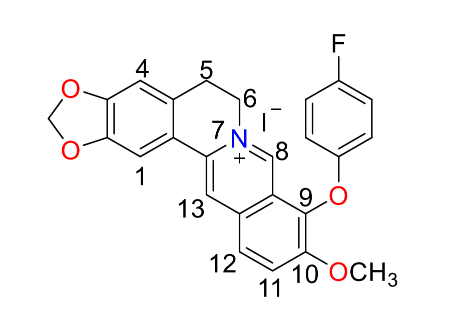

3l, yellow solid, m.p. 232-234 °C, yield 97%. ^{1}H NMR (300 MHz, DMSO-d_6): δ 9.83 (s, 1 H, H-8), 9.06 (s, 1 H, H-13), 8.31 (d, 1 H, J=9.2 Hz, H-12), 8.20 (d, 1 H, J=9.2 Hz, H-11), 7.83 (s, 1 H, H-1), 7.19 (t, 2 H, J=8.9 Hz, Ar-H), 7.09 (s, 1 H, H-4), 7.02-6.98 (m, 2 H, Ar-H), 6.18 (s, 2 H, OCH_2O), 4.92 (br-s, 2 H, H-6), 3.92 (s, 3 H, OCH_3), 3.19 (br-s, 2 H, H-5); $^{13}C\{^{1}H\}$ NMR (75 MHz, DMSO-d_6): δ 157.7 (d, J=237.5 Hz), 153.9 (d, J=1.8 Hz), 150.7, 150.0, 147.7, 144.6, 138.1, 137.2, 133.4, 130.8, 126.7, 125.9, 121.9, 120.6, 120.4, 117.1 (d, J=8.3 Hz), 116.1 (d, J=23.5 Hz), 108.4, 105.5 (Ar-C), 102.2 (OCH_2O), 57.1 (OCH_3), 55.3 (C-6), 26.2 (C-5); ^{19}F NMR (282 MHz, $CDCl_3$): δ -121.2; HRMS (ESI): m/z for $C_{25}H_{19}FNO_4^+$ $[M - I]^+$ calcd. 416.1293, found 416.1298.

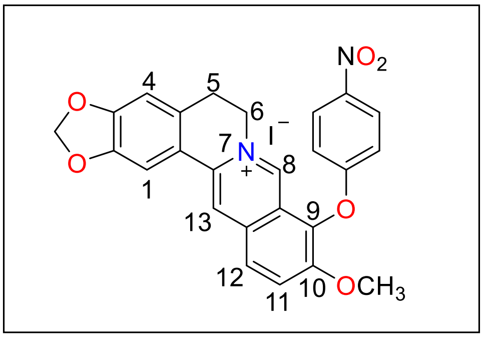

3m, brownish yellow solid, m.p. 245-247 °C, yield 80%. ^{1}H NMR (300 MHz, DMSO-d_6): δ 9.87 (s, 1 H, H-8), 9.10 (s, 1 H, H-13), 8.37 (d, 1 H, J=9.2 Hz, H-12), 8.29-8.24 (m, 3 H, H-11 & Ar-H), 7.83 (s, 1 H, H-1), 7.23-7.17 (m, 2 H, Ar-H), 7.09 (s, 1 H, H-4), 6.18 (s, 2 H, OCH_2O), 4.90 (t, 2 H, J=6.0 Hz, H-6), 3.96 (s, 3 H, OCH_3), 3.19 (t, 2 H, J=6.0 Hz, H-5); $^{13}C\{^{1}H\}$ NMR (75 MHz, DMSO-d_6): δ 162.1, 150.5, 150.0, 147.7, 144.4, 142.6, 138.3, 135.4, 133.4, 130.9, 126.7, 126.5, 125.9, 121.4, 120.7, 120.3, 116.4, 108.4, 105.5 (Ar-C), 102.2 (OCH_2O), 57.2 (OCH_3), 55.3 (C-6), 26.2 (C-5); HRMS (ESI): m/z for $C_{25}H_{19}N_2O_6^+$ $[M - I]^+$ calcd. 443.1238, found 443.1247.

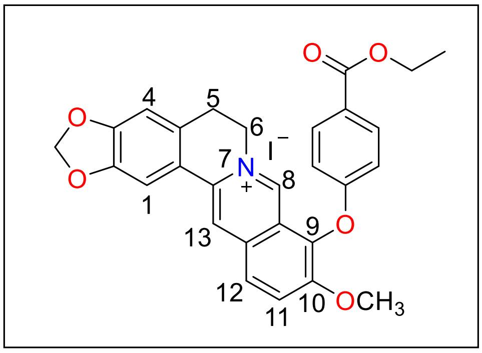

3n, yellow solid, m.p. 258-260 °C, yield 75%. ^{1}H NMR (300 MHz, DMSO-d_6): δ 9.84 (s, 1 H, H-8), 9.08 (s, 1 H, H-13), 8.34 (d, 1 H, J=9.2 Hz, H-12), 8.24 (d, 1 H, J=9.2 Hz, H-11), 7.96 (d, 2 H, J=8.7 Hz, Ar-H), 7.83 (s, 1 H, H-1), 7.08 (s, 2 H, Ar-H), 7.06 (s, 1 H, H-4), 6.18 (s, 2 H, OCH_2O), 4.90 (br-s, 2 H, H-6), 4.30 (q, 2 H, J=6.9 Hz, CH_2), 3.93 (s, 3 H, OCH_3), 3.19 (br-s, 2 H, H-5), 1.30 (t, 3 H, J=6.9 Hz, CH_3); $^{13}C\{^{1}H\}$ NMR (75 MHz, DMSO-d_6): δ 165.1 (CO), 161.0, 150.6, 150.0, 147.7, 144.5, 138.3, 136.1, 133.4, 131.3, 130.9, 126.6, 126.3, 124.5, 121.6, 120.7, 120.3, 115.7, 108.4, 105.5 (Ar-C), 102.2 (OCH_2O), 60.6 (CH_2), 57.1 (OCH_3), 55.3 (C-6), 26.2

(C-5), 14.2 (CH_3); HRMS (ESI): *m/z* for $C_{28}H_{24}NO_6^+$ $[M - I]^+$ calcd. 470.1598, found 470.1605.

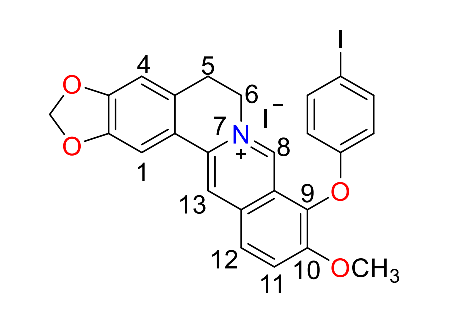

3o, yellow solid, m.p. 251-253 °C, yield 81%. ^{1}H NMR (300 MHz, DMSO-d_6): δ 9.79 (s, 1 H, H-8), 9.06 (s, 1 H, H-13), 8.31 (d, 1 H, J=9.2 Hz, H-12), 8.21 (d, 1 H, J=9.2 Hz, H-11), 7.82 (s, 1 H, H-1), 7.66 (d, 2 H, J=8.9 Hz, Ar-H), 7.08 (s, 1 H, H-4), 6.80 (d, 2 H, J=8.9 Hz, Ar-H), 6.18 (s, 2 H, OCH_2O), 4.90 (br-s, 2 H, Hz, H-6), 3.93 (s, 3 H, OCH_3), 3.18 (br-s, 2 H, J=6.0 Hz, H-5); $^{13}C\{^1H\}$ NMR (75 MHz, DMSO-d_6): δ 157.6, 150.7, 150.0, 147.7, 144.6, 138.23, 138.18, 136.4, 133.4, 130.9, 126.6, 126.1, 121.8, 120.7, 120.4, 118.2, 108.5, 105.5 (Ar-C), 102.2 (OCH_2O), 86.2 (Ar-C), 57.1 (OCH_3), 55.3 (C-6), 26.2 (C-5); HRMS (ESI): *m/z* for $C_{25}H_{19}INO_4^+$ $[M - I]^+$ calcd. 524.0353, found 524.0361.

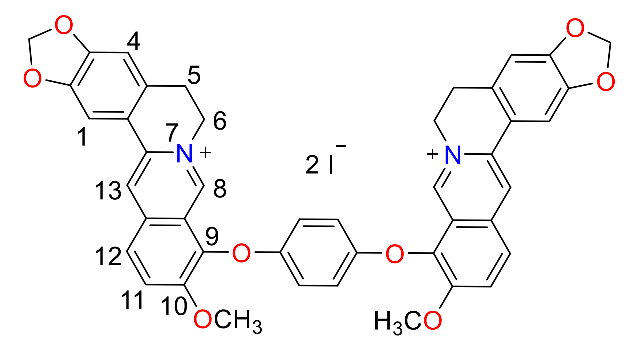

5, yellow solid, m.p. > 300 °C, yield 68%. ^{1}H NMR (300 MHz, DMSO-d_6): δ 9.85 (s, 2 H, H-8), 9.06 (s, 2 H, H-13), 8.31 (d, 2 H, J=9.2 Hz, H-12), 8.22 (d, 2 H, J=9.2 Hz, H-11), 7.83 (s, 2 H, H-1), 7.10 (s, 2 H, H-4), 6.95 (s, 4 H, Ar-H), 6.18 (s, 4 H, OCH_2O), 4.92 (br-s, 4 H, H-6), 3.93 (s, 6 H, OCH_3), 3.19 (br-s, 4 H, H-5); Due to

the poor solubility, the ^{13}C spectrum only showed few signals even with long acquisition time. HRMS (ESI): *m/z* for $C_{44}H_{34}N_2O_8^{2+}$ $[M - 2I]^{2+}$ calcd. 359.1152, found 359.1159.

NMR spectra

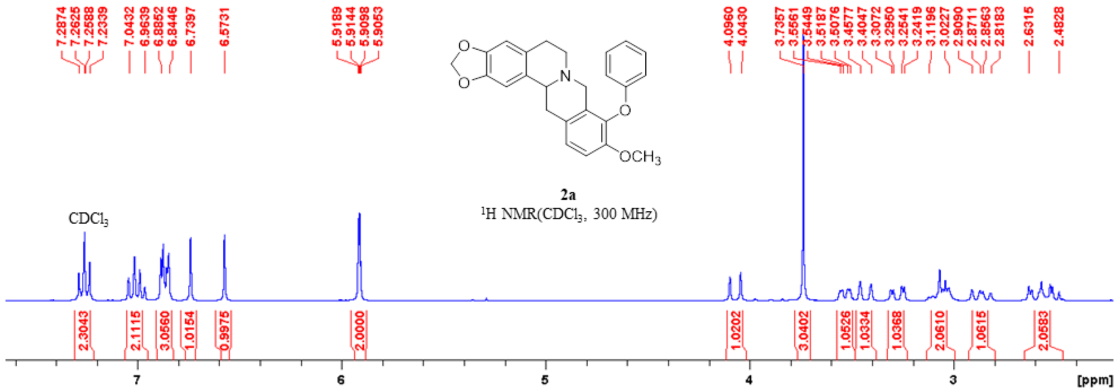
2a
^{1}H NMR(CDCl$_3$, 300 MHz)
CDCl$_3$
OCH$_3$
[ppm]

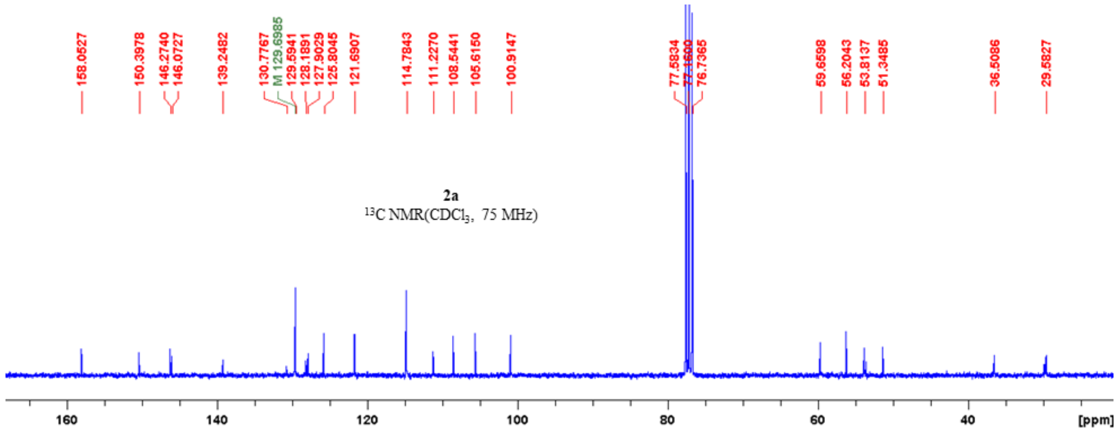
2a
^{13}C NMR(CDCl$_3$, 75 MHz)
[ppm]

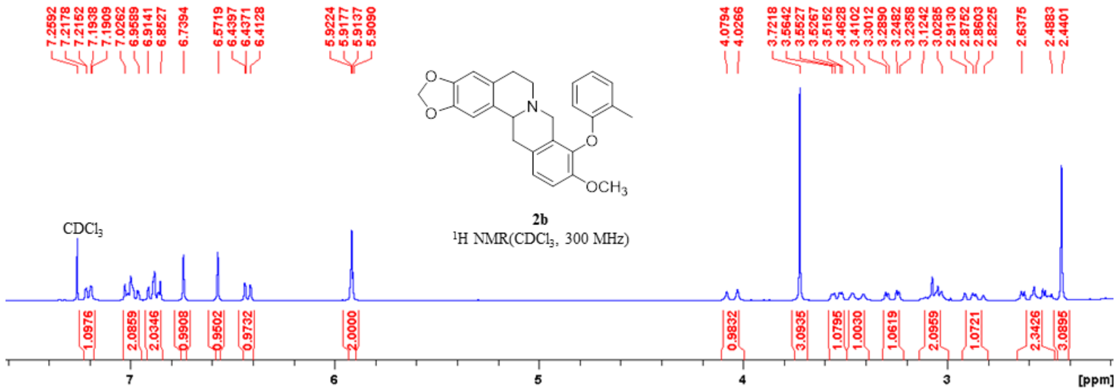
CDCl$_3$
2b
^{1}H NMR(CDCl$_3$, 300 MHz)
OCH$_3$
[ppm]

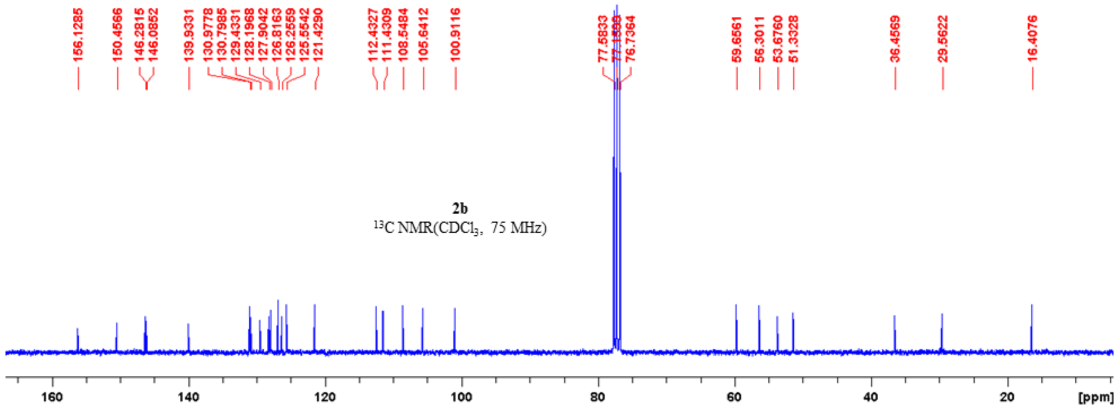
2b
^{13}C NMR(CDCl$_3$, 75 MHz)
[ppm]

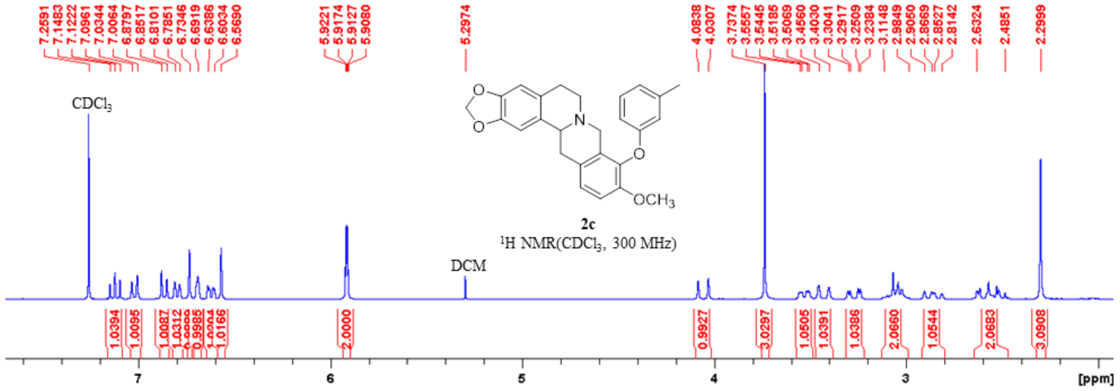
CDCl3
DCM
OCH3
2c
1H NMR(CDCl3, 300 MHz)
[ppm]

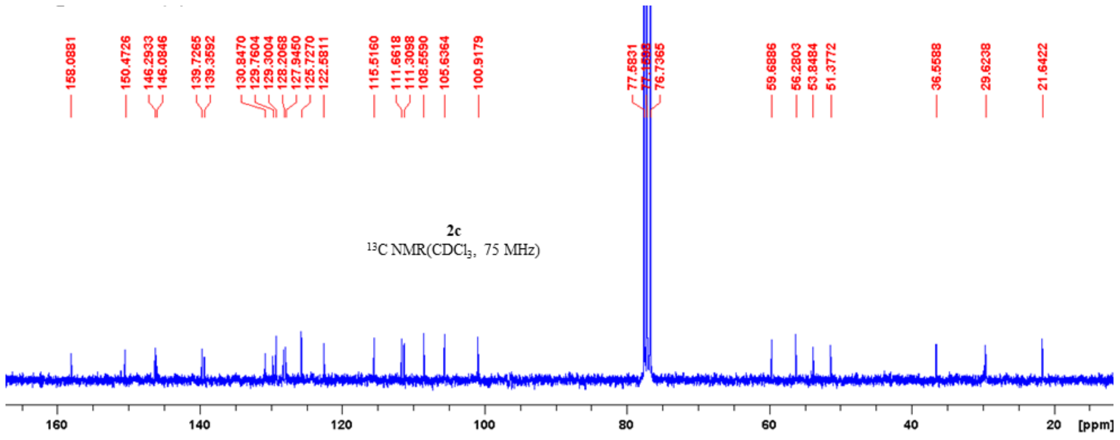
2c
13C NMR(CDCl3, 75 MHz)
[ppm]

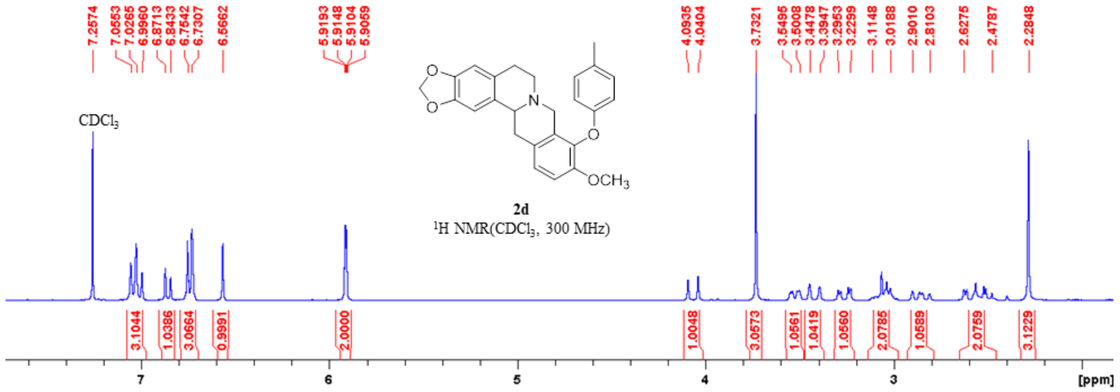
CDCl3
2d
1H NMR(CDCl3, 300 MHz)
OCH3

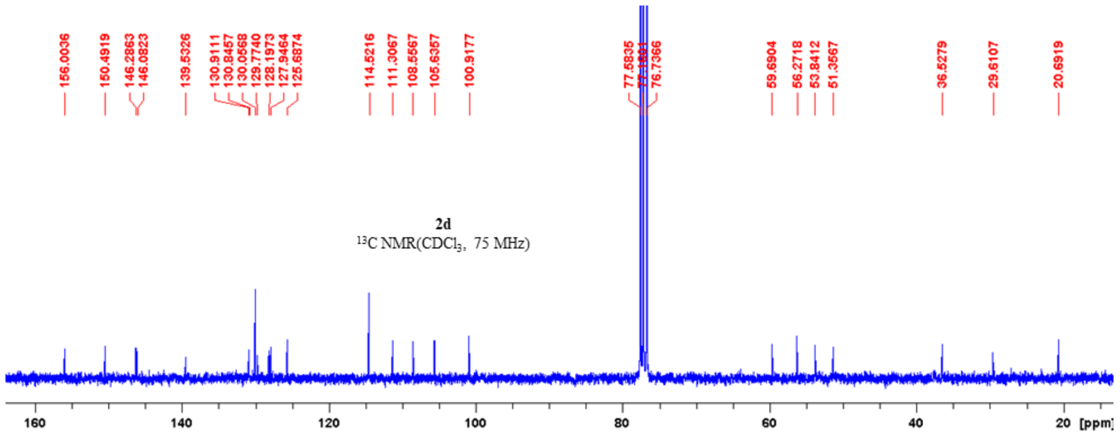
2d
13C NMR(CDCl3, 75 MHz)

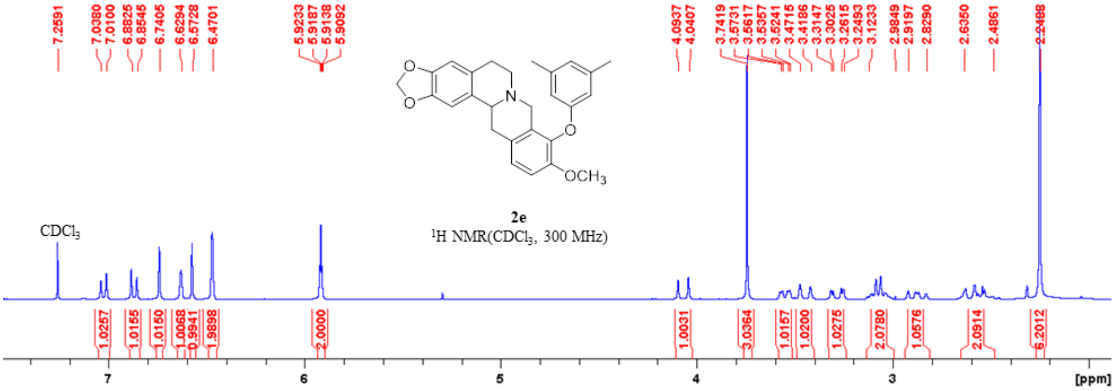
CDCl$_3$
2e
^{1}H NMR(CDCl$_3$, 300 MHz)
OCH$_3$
[ppm]

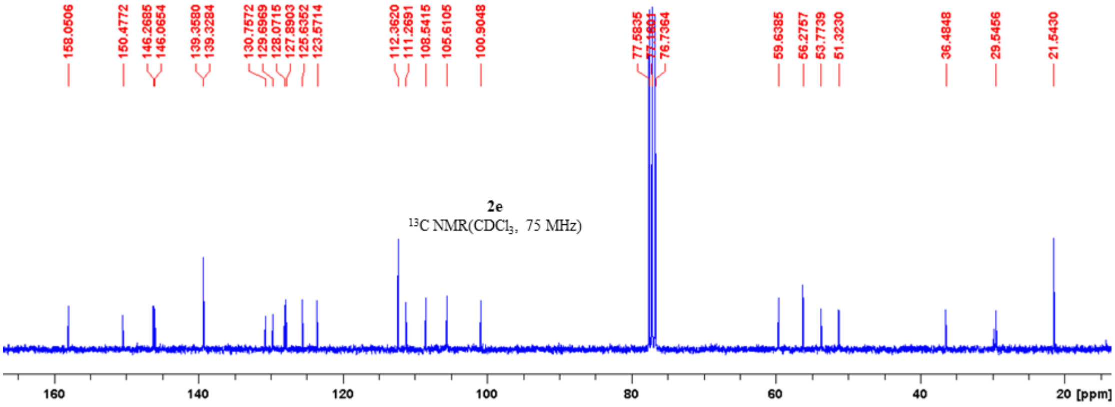
2e
^{13}C NMR(CDCl$_3$, 75 MHz)
[ppm]

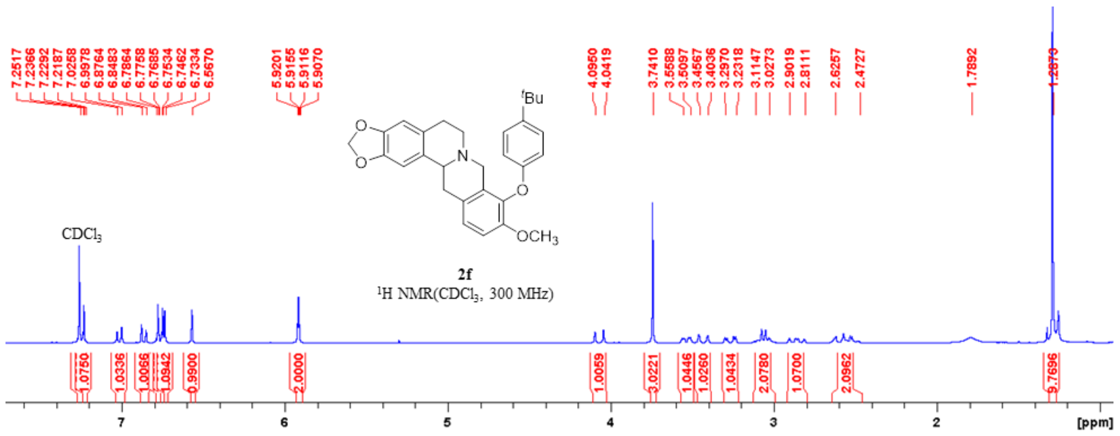
CDCl3
2f
1H NMR(CDCl3, 300 MHz)
[ppm]

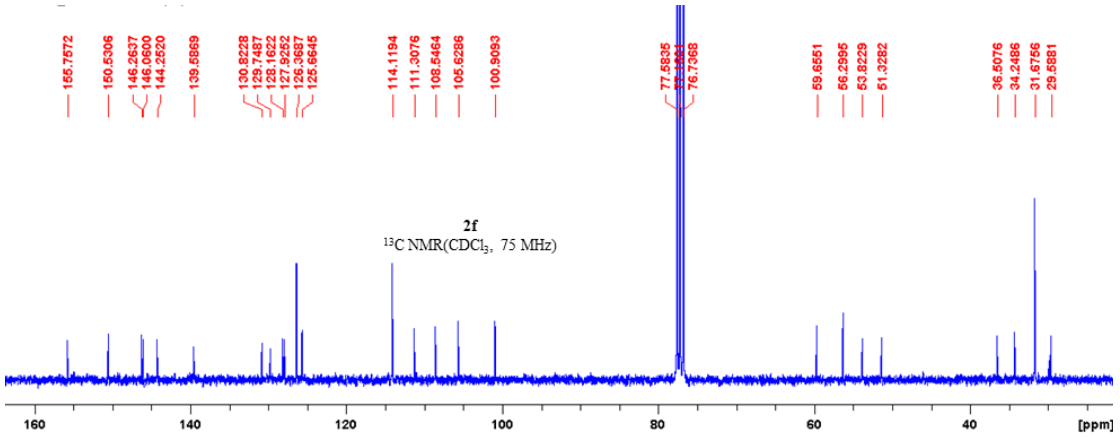
2f
13C NMR(CDCl3, 75 MHz)
[ppm]

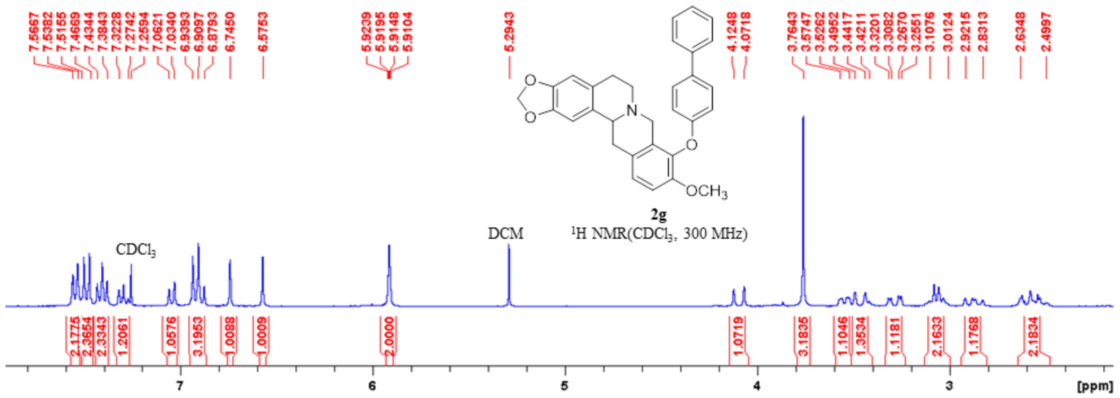
2g
1H NMR(CDCl3, 300 MHz)
CDCl3
DCM
[ppm]

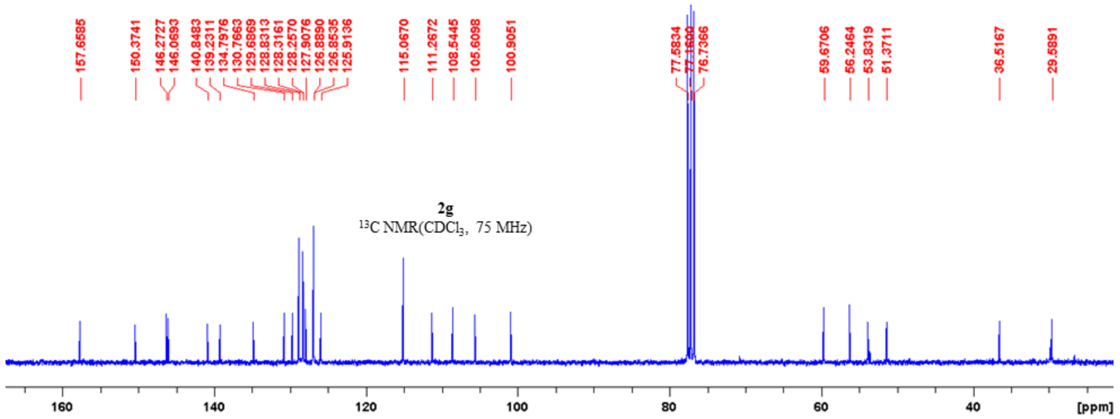
2g
13C NMR(CDCl3, 75 MHz)
[ppm]

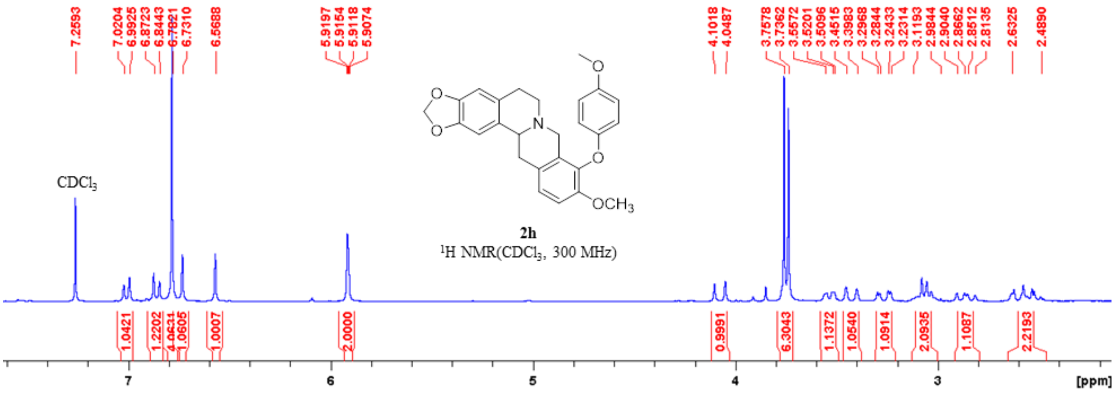
CDCl3
OCH3
2h
1H NMR(CDCl3, 300 MHz)
[ppm]

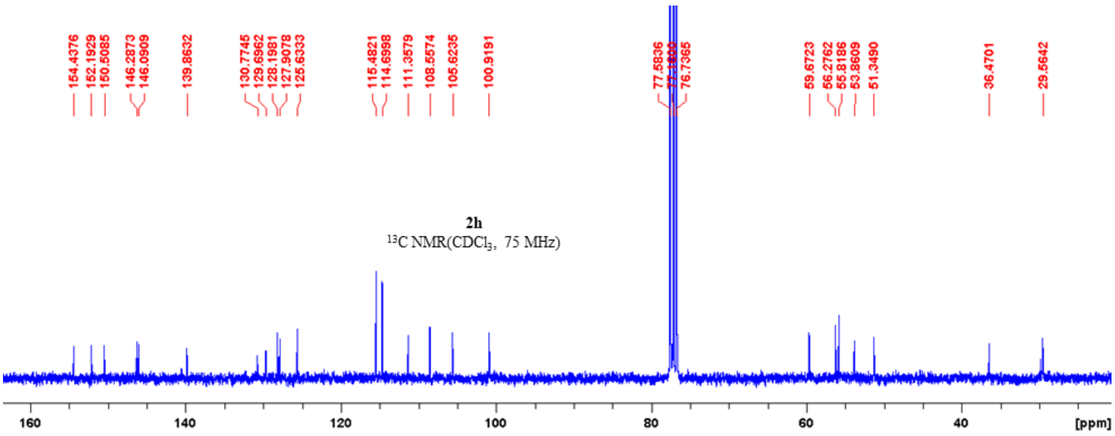
2h
13C NMR(CDCl3, 75 MHz)
[ppm]

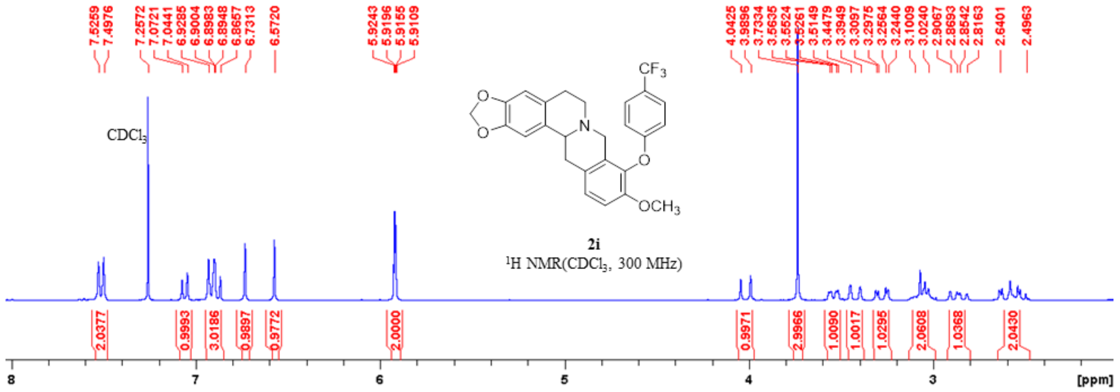
CDCl3
CF3
N
O
OCH3
2i
1H NMR(CDCl3, 300 MHz)
[ppm]

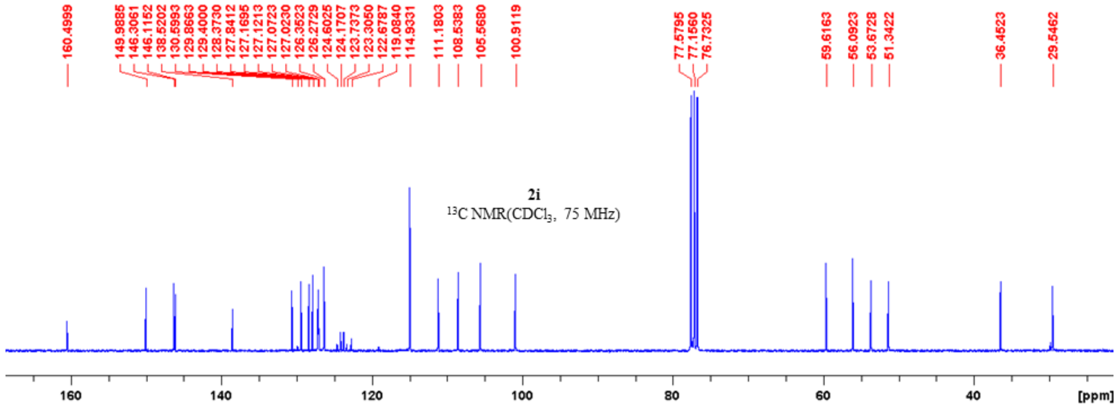
2i
13C NMR(CDCl3, 75 MHz)
[ppm]

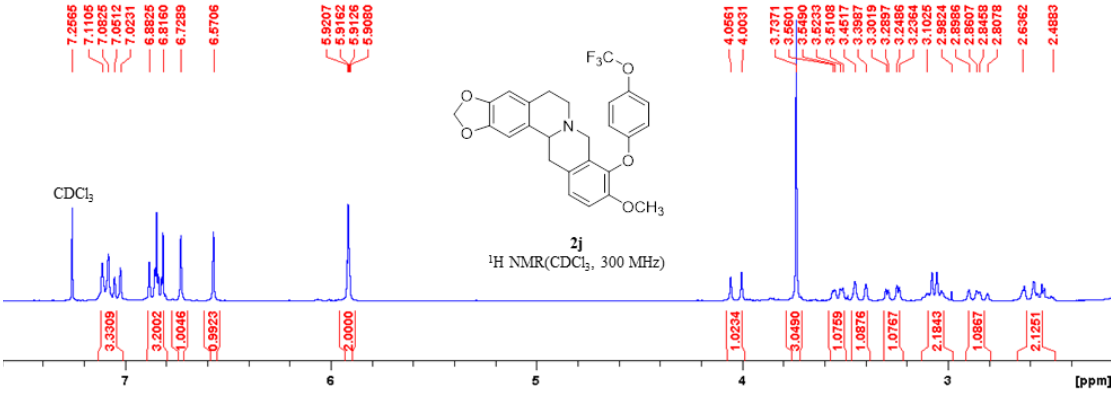

2j

^{1}H NMR(CDCl$_3$, 300 MHz)

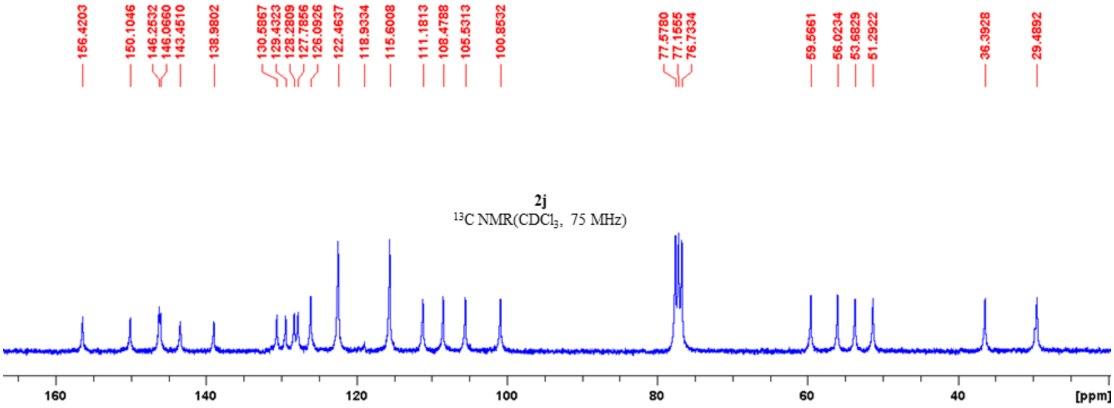

2j

^{13}C NMR(CDCl$_3$, 75 MHz)

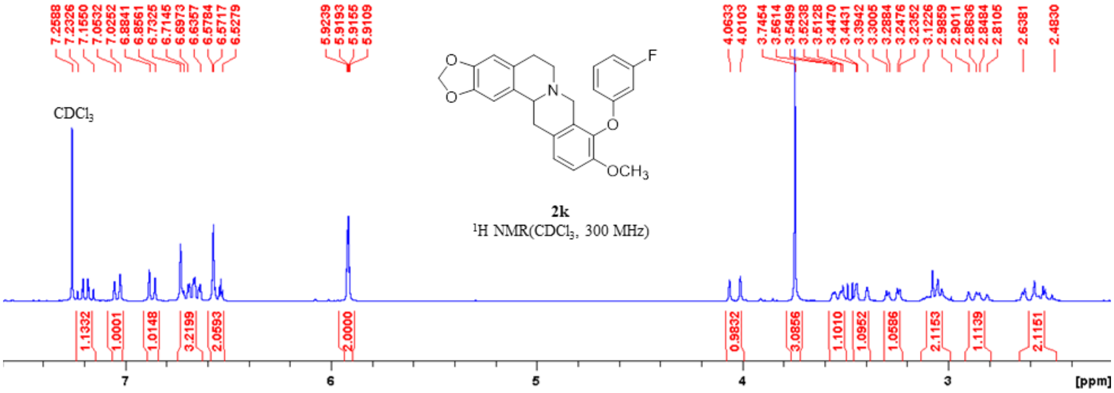

2k

^{1}H NMR(CDCl$_3$, 300 MHz)

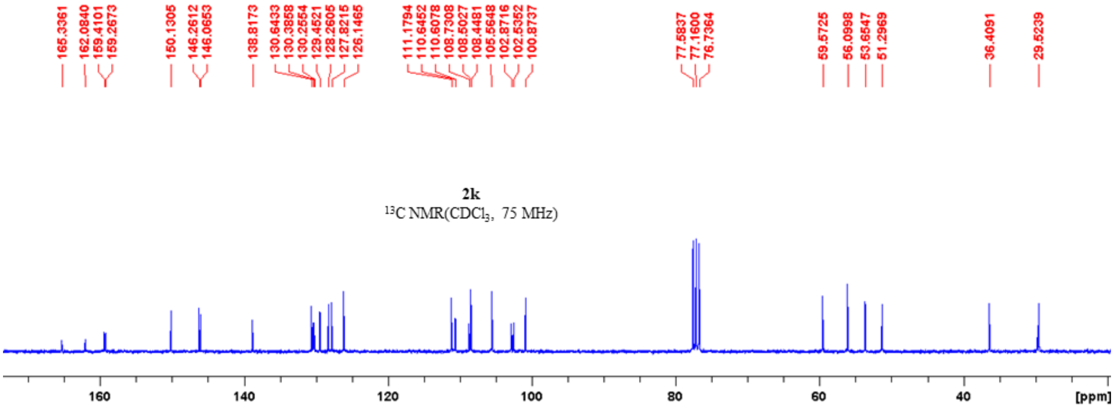

2k

^{13}C NMR(CDCl$_3$, 75 MHz)

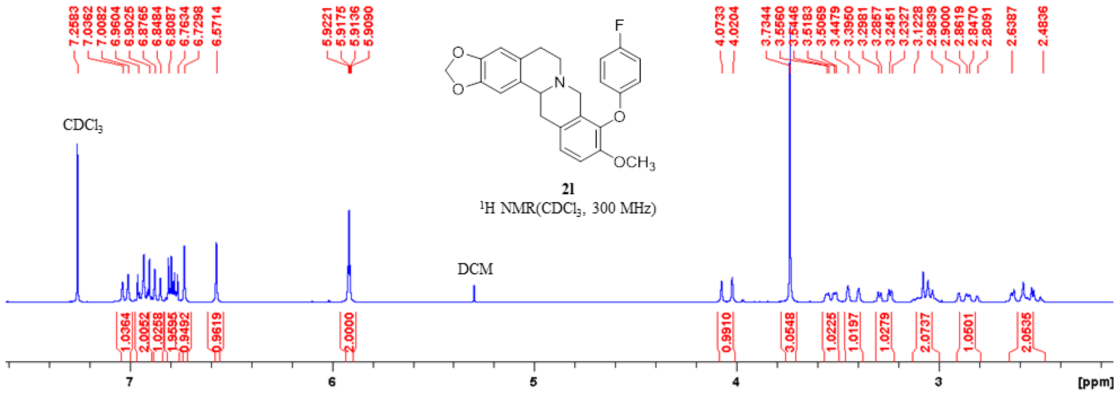

21

^{1}H NMR(CDCl$_3$, 300 MHz)

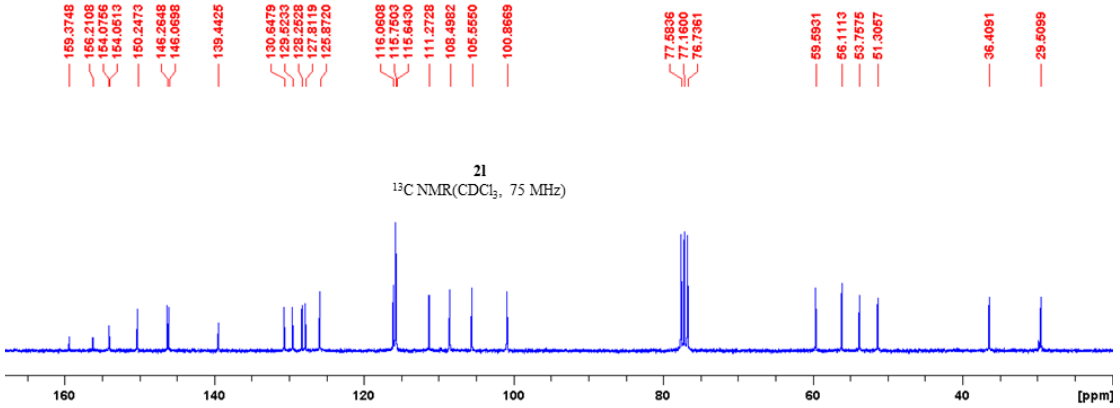

21

^{13}C NMR(CDCl$_3$, 75 MHz)

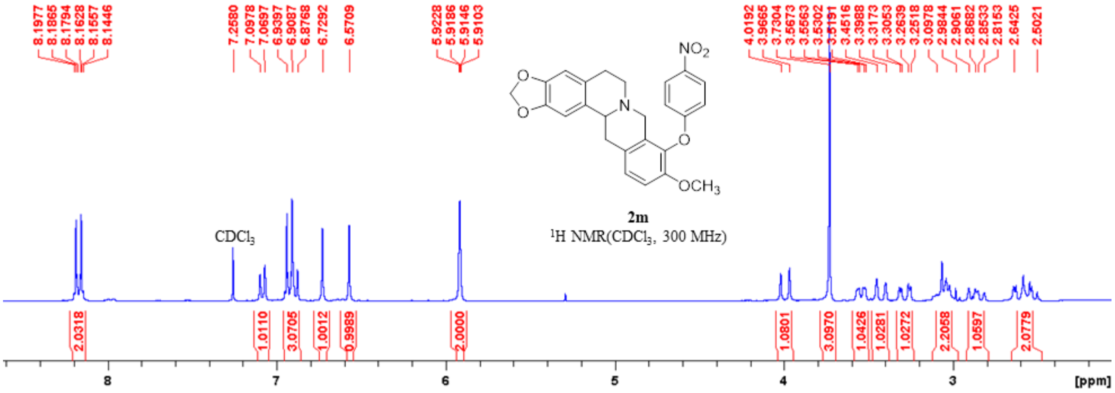

2m
^{1}H NMR(CDCl$_3$, 300 MHz)
CDCl$_3$
[ppm]

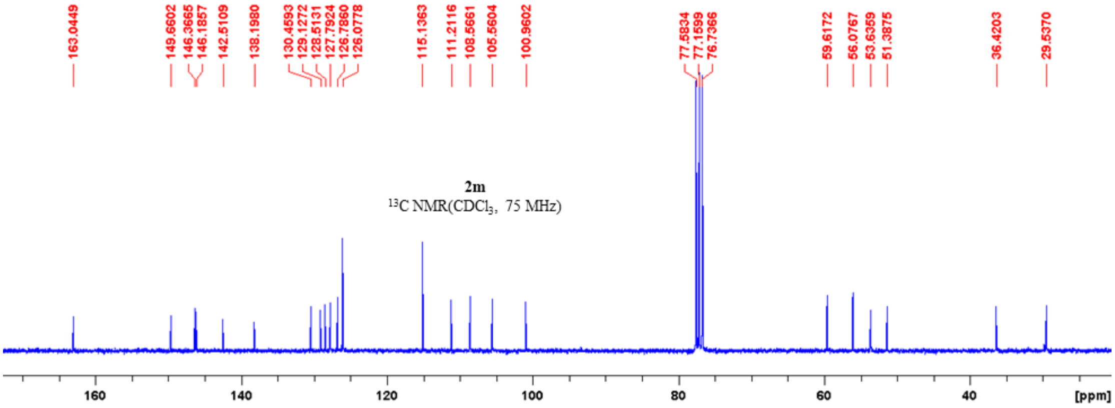

2m
^{13}C NMR(CDCl$_3$, 75 MHz)
[ppm]

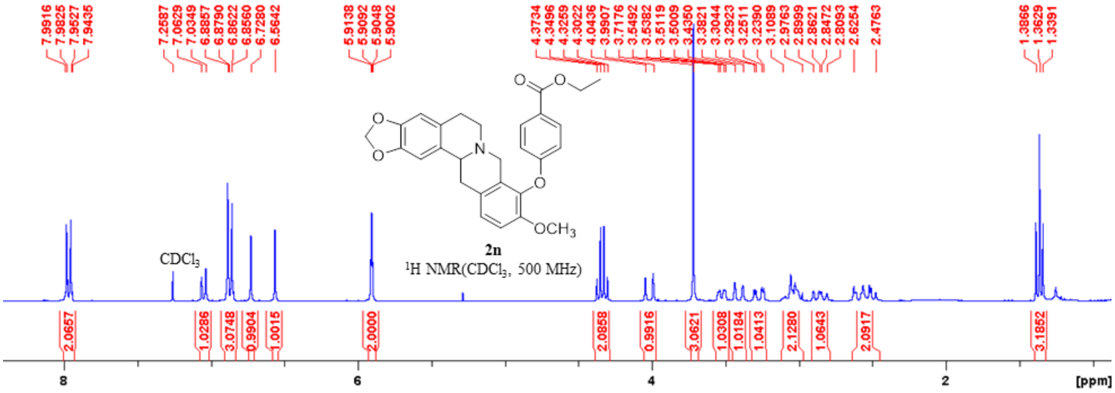
CDCl3
OCH3
2n
1H NMR(CDCl3, 500 MHz)
[ppm]

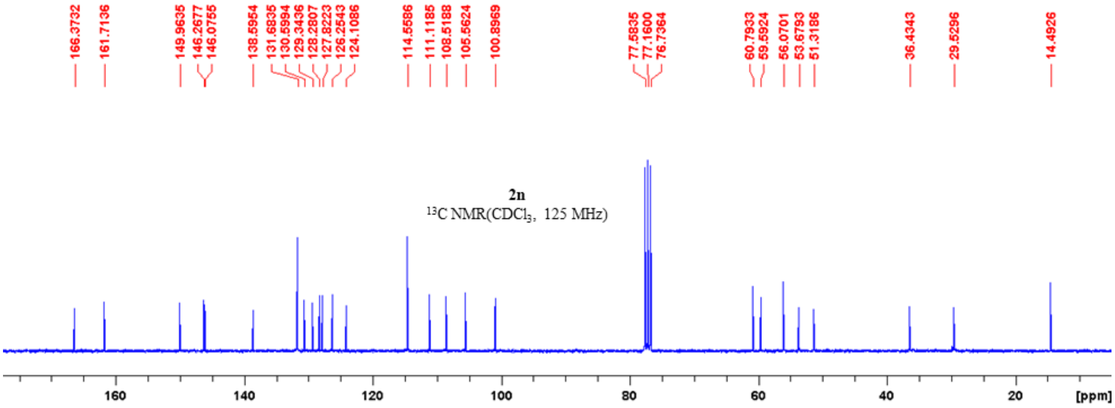
2n
13C NMR(CDCl3, 125 MHz)
[ppm]

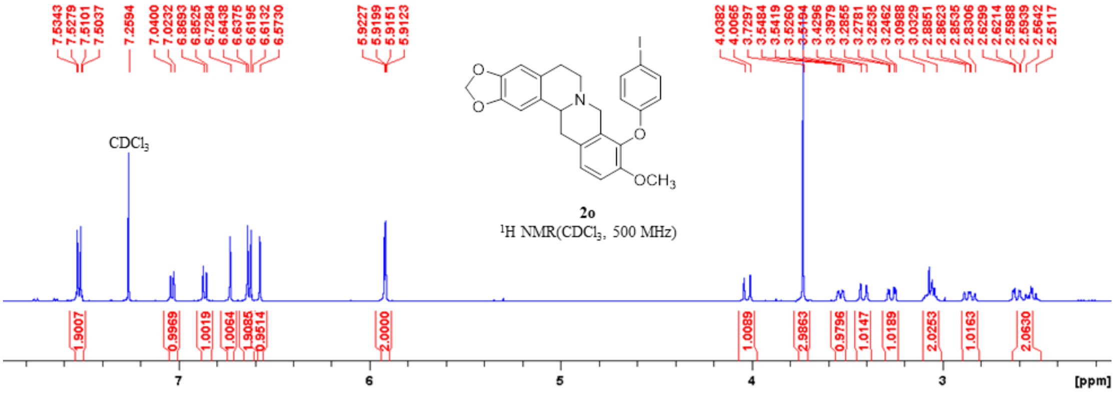
7.5343
7.5279
7.5101
7.5037
7.2594
7.0400
7.0232
6.8693
6.8525
6.7284
6.6438
6.6375
6.6195
6.6132
6.5730
5.9227
5.9199
5.9151
5.9123
4.0382
4.0065
3.7297
3.5484
3.5419
3.5260
3.5194
3.4296
3.3979
3.2855
3.2781
3.2535
3.2462
3.0988
3.0329
2.8851
2.8623
2.8535
2.8306
2.6299
2.6214
2.5988
2.5939
2.5642
2.5117
CDCl3
OCH3
2o
1H NMR(CDCl3, 500 MHz)
1.9007
0.9969
1.0019
1.0064
1.9085
0.9514
2.0000
1.0089
2.9863
0.9796
1.0147
1.0189
2.0253
1.0163
2.0630
7
6
5
4
3
[ppm]

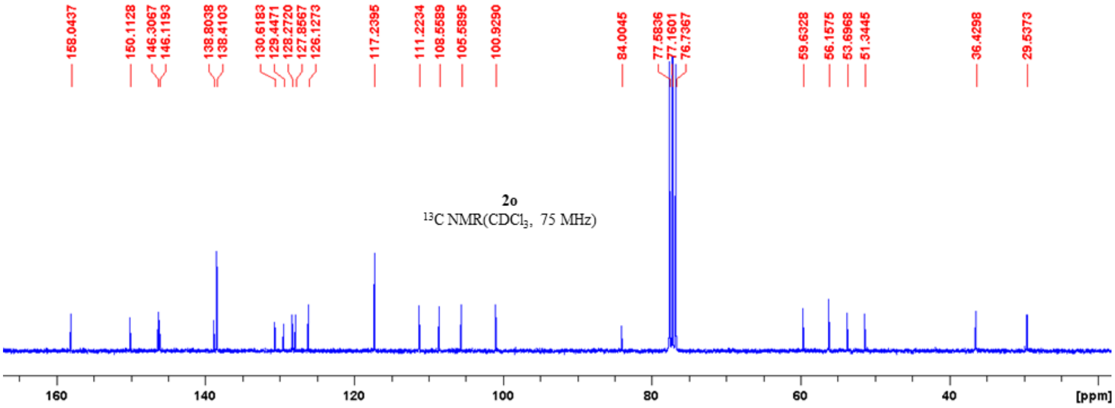
158.0437
150.1128
146.3067
146.1193
138.8038
138.4103
130.6183
129.4471
128.2720
127.8567
126.1273
117.2395
111.2234
108.5589
105.5895
100.9290
84.0045
77.5836
77.1601
76.7367
59.6328
56.1575
53.6968
51.3445
36.4298
29.5373
2o
13C NMR(CDCl3, 75 MHz)
160
140
120
100
80
60
40
[ppm]

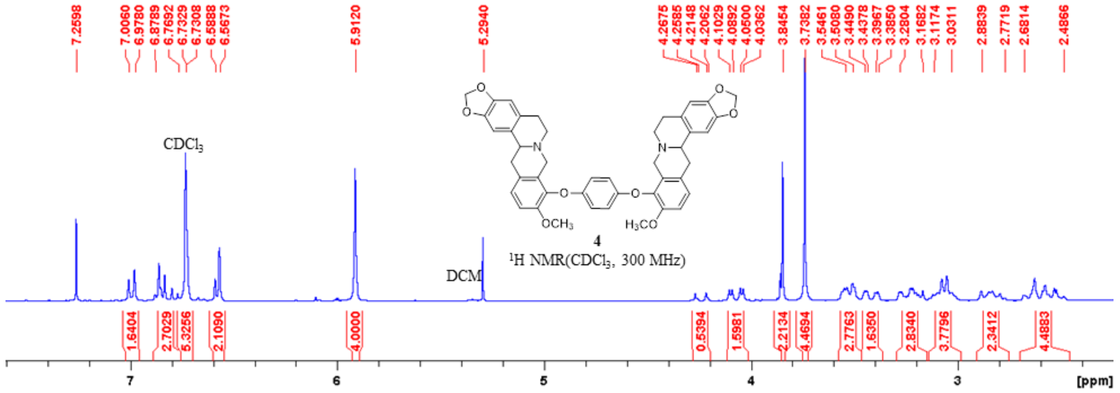
CDCl3
DCM
OCH3
H3CO
4
1H NMR(CDCl3, 300 MHz)
[ppm]

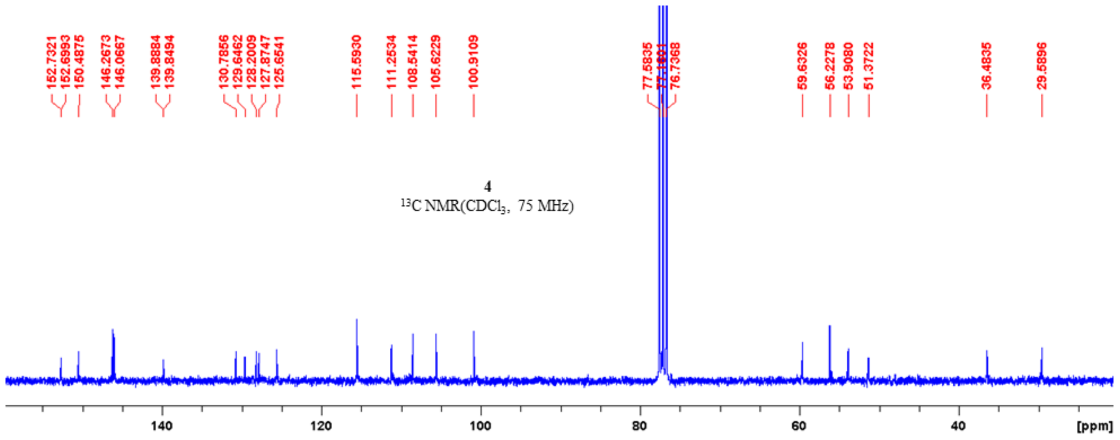
4
13C NMR(CDCl3, 75 MHz)
[ppm]

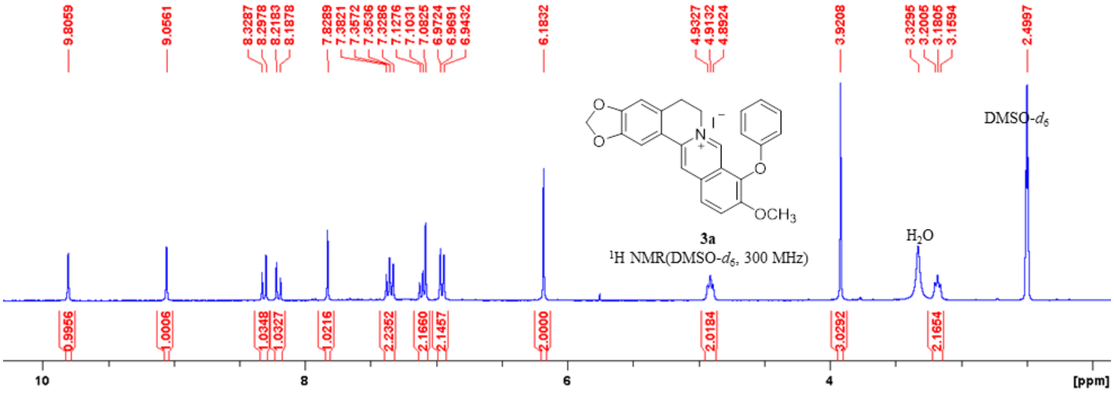

3a

^{1}H NMR(DMSO-d_6, 300 MHz)

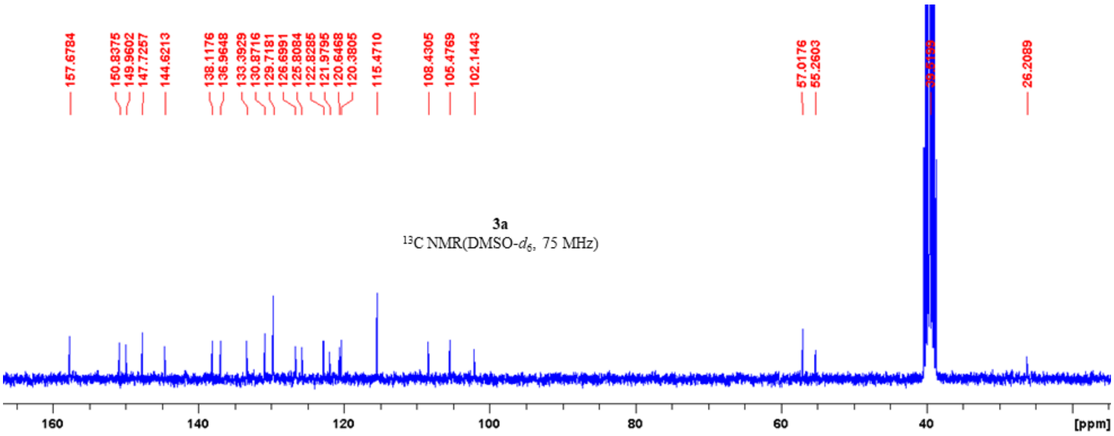

3a

^{13}C NMR(DMSO-d_6, 75 MHz)

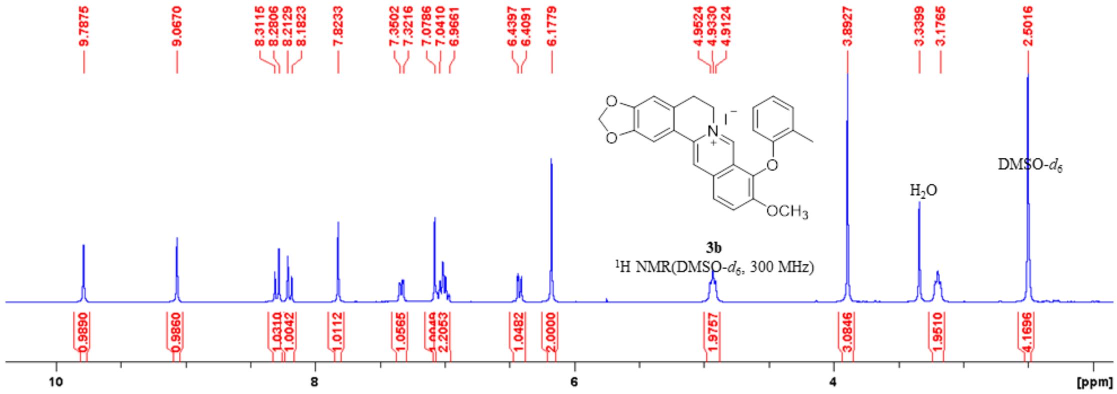
9.7875
9.0670
8.3115
8.2806
8.2129
8.1823
7.8233
7.3502
7.3216
7.0786
7.0410
6.9661
6.4397
6.4091
6.1779
4.9524
4.9330
4.9124
3.8927
3.3399
3.1765
2.5016
OCH3
DMSO-d6
H2O
3b
1H NMR(DMSO-d6, 300 MHz)
0.9890
0.9860
1.0310
1.0042
1.0112
1.0565
2.2053
1.0482
2.0000
1.9757
3.0846
1.9510
4.1696
10
8
6
4
[ppm]

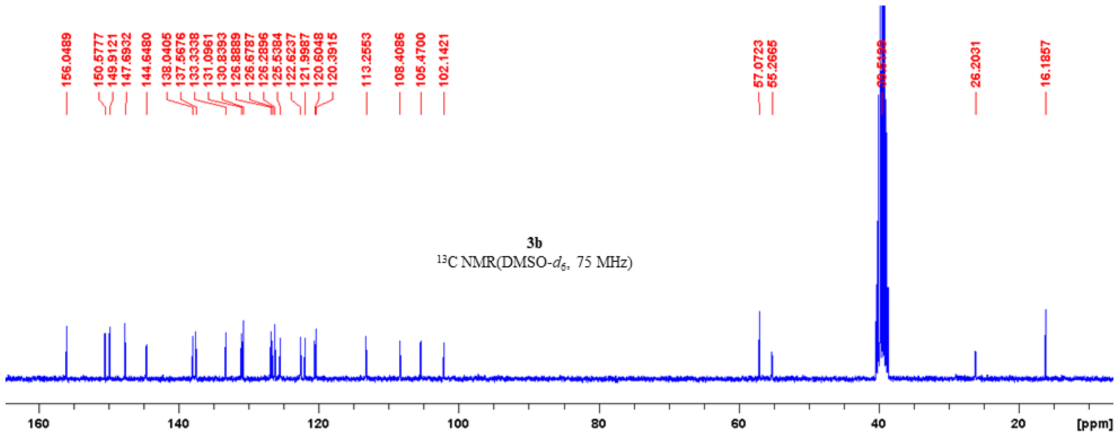
156.0489
150.5777
149.9121
147.6932
144.6480
138.0405
137.5676
133.3338
131.0961
130.8393
126.8889
126.6787
126.2896
125.5384
122.6237
121.9987
120.6048
120.3915
113.2553
108.4086
105.4700
102.1421
57.0723
55.2665
26.2031
16.1857
3b
13C NMR(DMSO-d6, 75 MHz)
160
140
120
100
80
60
40
20
[ppm]

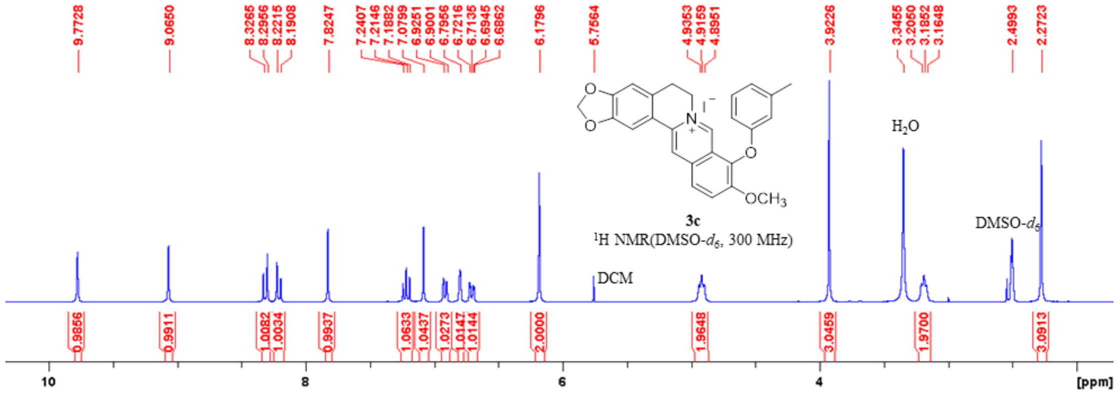

9.7728
9.0650
8.3265
8.2956
8.2215
8.1908
7.8247
7.2407
7.2146
7.1882
7.0799
6.9251
6.9001
6.7956
6.7216
6.7135
6.6945
6.6862
6.1796
5.7564
4.9353
4.9159
4.8951
3.9226
3.3455
3.2050
3.1852
3.1648
2.4993
2.2723
3c
^1H NMR(DMSO-d_6, 300 MHz)
DCM
H_2O
DMSO-d_6
OCH3
0.9856
0.9911
1.0082
1.0034
0.9937
1.0633
1.0437
1.0273
1.0147
1.0144
2.0000
1.9648
3.0459
1.9700
3.0913
10
8
6
4
[ppm]

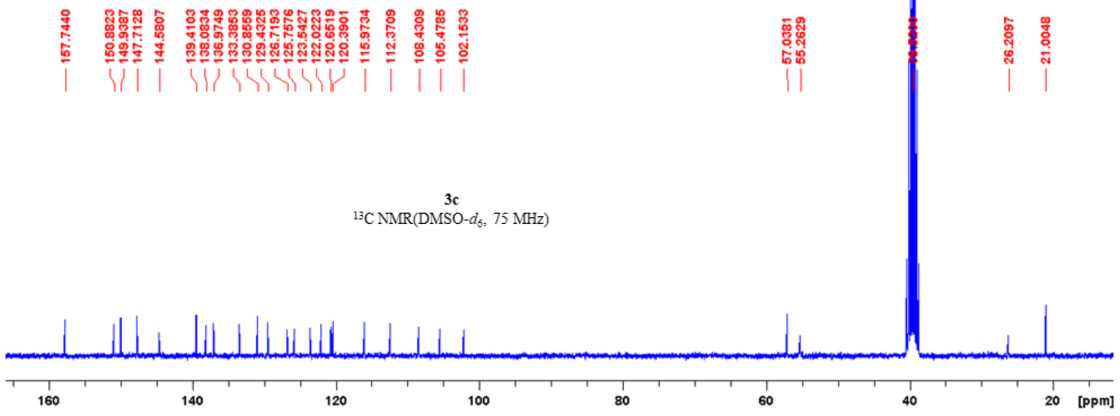

157.7440
150.8823
149.9387
147.7128
144.5807
139.4103
138.0834
136.9749
133.3853
130.8559
129.4325
126.7193
125.7576
123.5427
122.0223
120.6519
120.3901
115.9734
112.3709
108.4309
105.4785
102.1533
57.0381
55.2629
26.2097
21.0048
3c
^{13}C NMR(DMSO-d_6, 75 MHz)
160
140
120
100
80
60
40
20
[ppm]

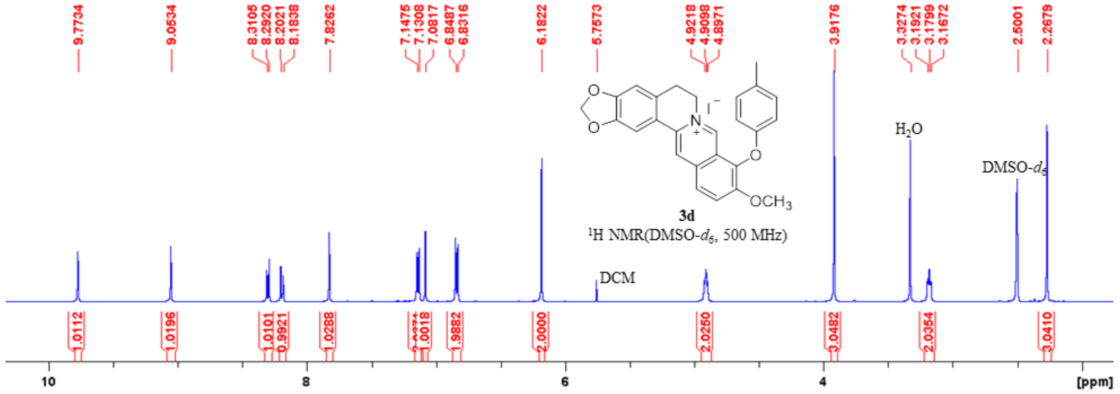
H$_2$O
DMSO-d_6
DCM
3d
^{1}H NMR(DMSO-d_6, 500 MHz)
[ppm]

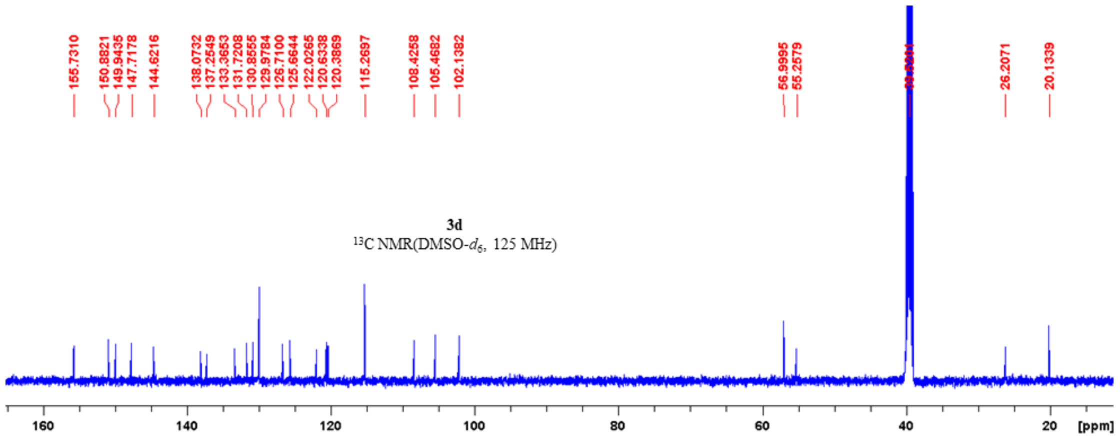
3d
^{13}C NMR(DMSO-d_6, 125 MHz)
[ppm]

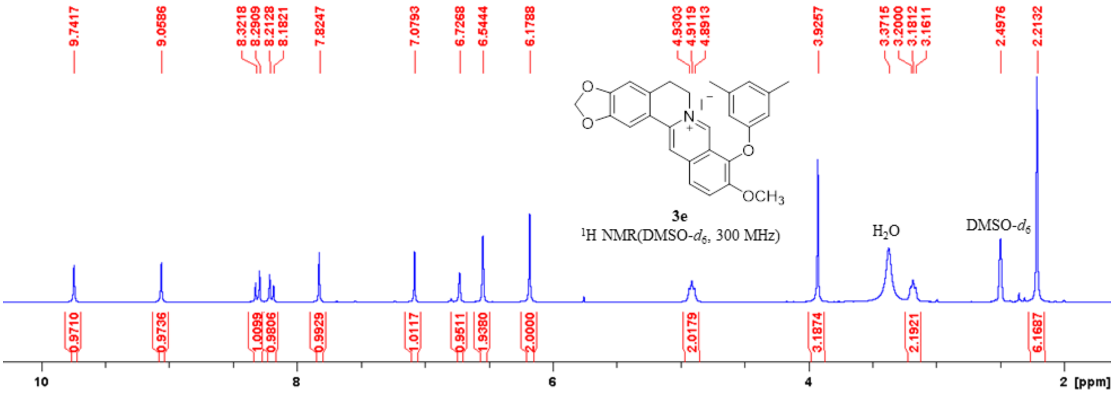
9.7417
9.0586
8.3218
8.2909
8.2128
8.1821
7.8247
7.0793
6.7268
6.5444
6.1788
4.9303
4.9119
4.8913
3.9257
3.3715
3.2000
3.1812
3.1611
2.4976
2.2132
OCH3
3e
1H NMR(DMSO-d6, 300 MHz)
H2O
DMSO-d6
0.9710
0.9736
1.0099
0.9806
0.9929
1.0117
0.9511
1.9380
2.0000
2.0179
3.1874
2.1921
6.1687
10
8
6
4
2 [ppm]

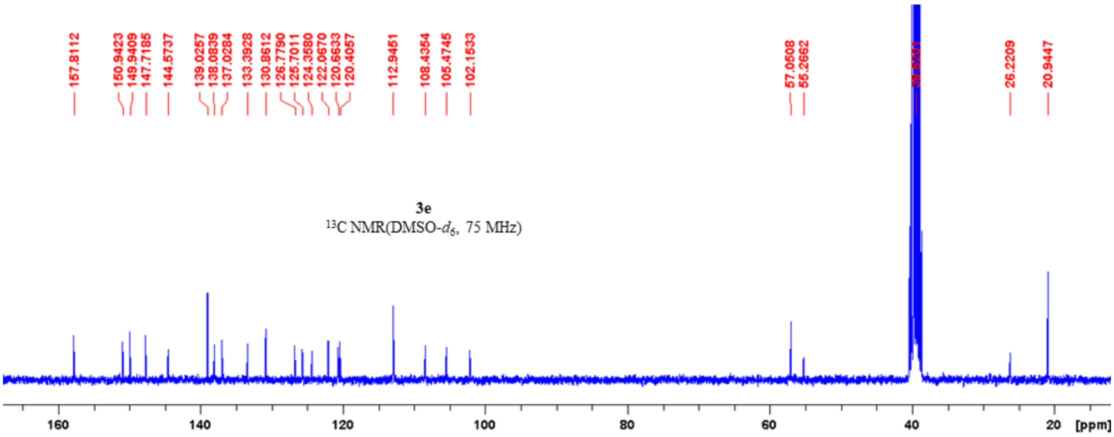
157.8112
150.9423
149.9409
147.7185
144.5737
139.0257
138.0839
137.0284
133.3928
130.8612
126.7790
125.7011
124.3580
122.0670
120.6633
120.4057
112.9451
108.4354
105.4745
102.1533
57.0508
55.2662
26.2209
20.9447
3e
13C NMR(DMSO-d6, 75 MHz)
160
140
120
100
80
60
40
20 [ppm]

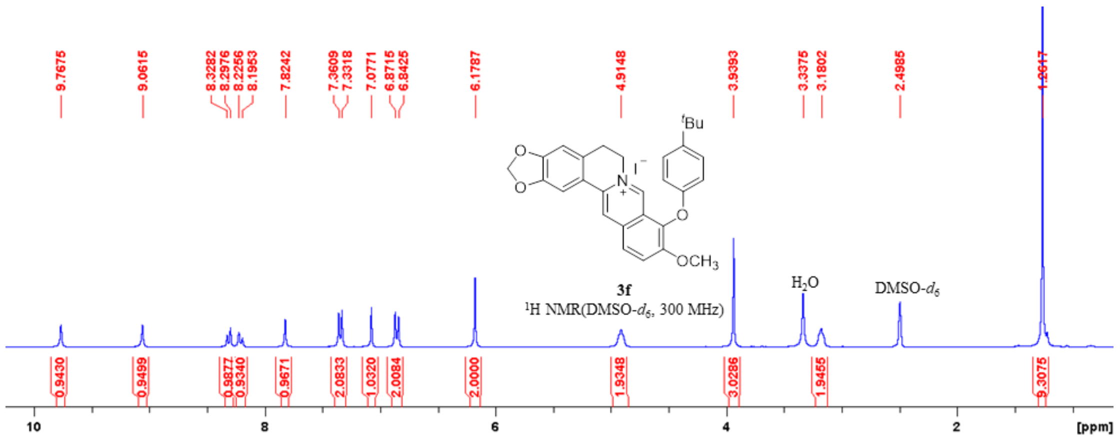
3f
1H NMR(DMSO-d6, 300 MHz)
H2O
DMSO-d6
tBu
OCH3

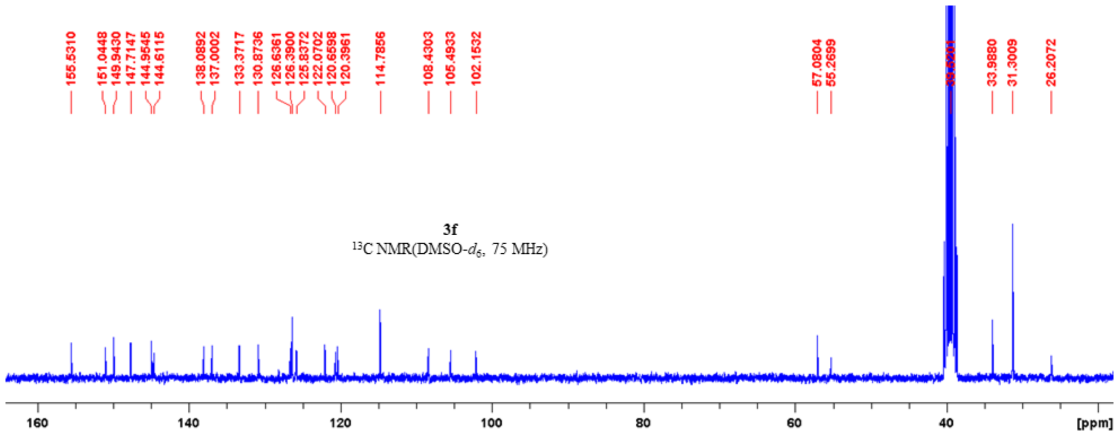
3f
13C NMR(DMSO-d6, 75 MHz)

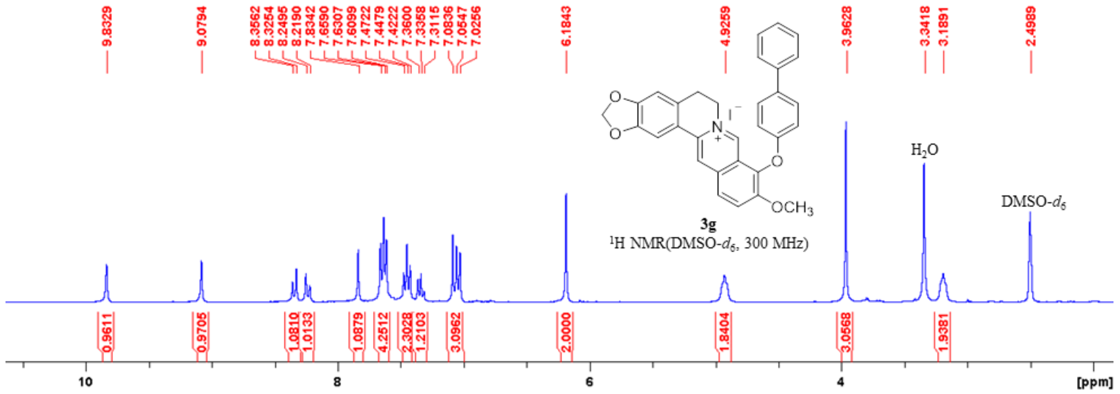
3g
^{1}H NMR(DMSO-d_6, 300 MHz)
H$_2$O
DMSO-d_6
[ppm]

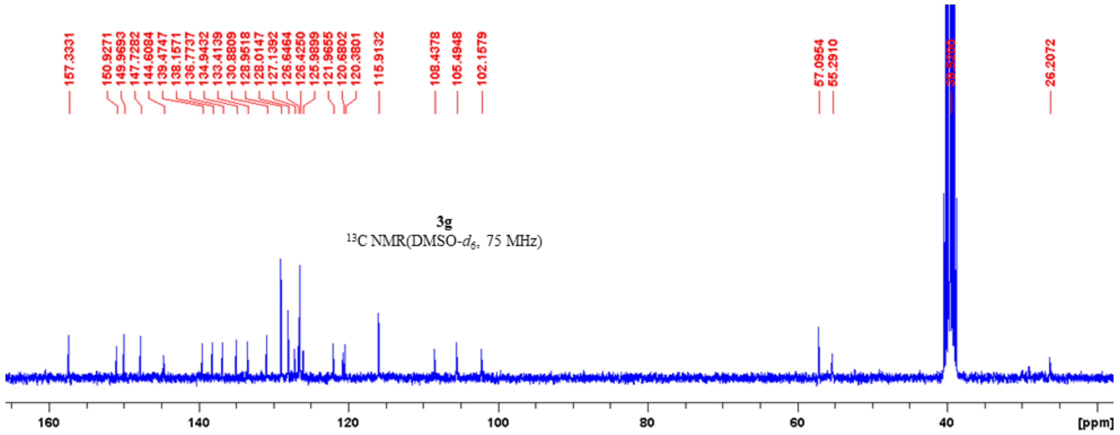
3g
^{13}C NMR(DMSO-d_6, 75 MHz)
[ppm]

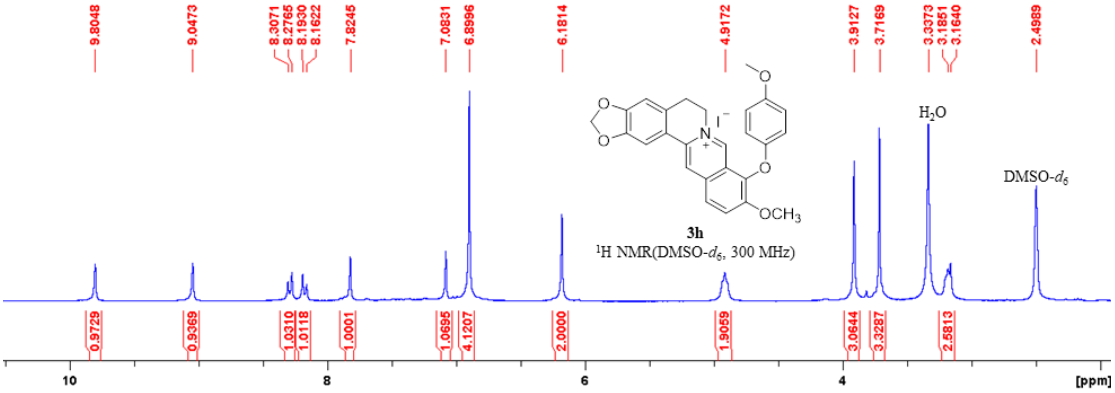
9.8048
9.0473
8.3071
8.2765
8.1930
8.1622
7.8245
7.0831
6.8996
6.1814
4.9172
3.9127
3.7169
3.3373
3.1851
3.1640
2.4989
H2O
DMSO-d6
OCH3
3h
1H NMR(DMSO-d6, 300 MHz)
0.9729
0.9369
1.0310
1.0118
1.0001
1.0695
4.1207
2.0000
1.9059
3.0644
3.3287
2.5813
10
8
6
4
[ppm]

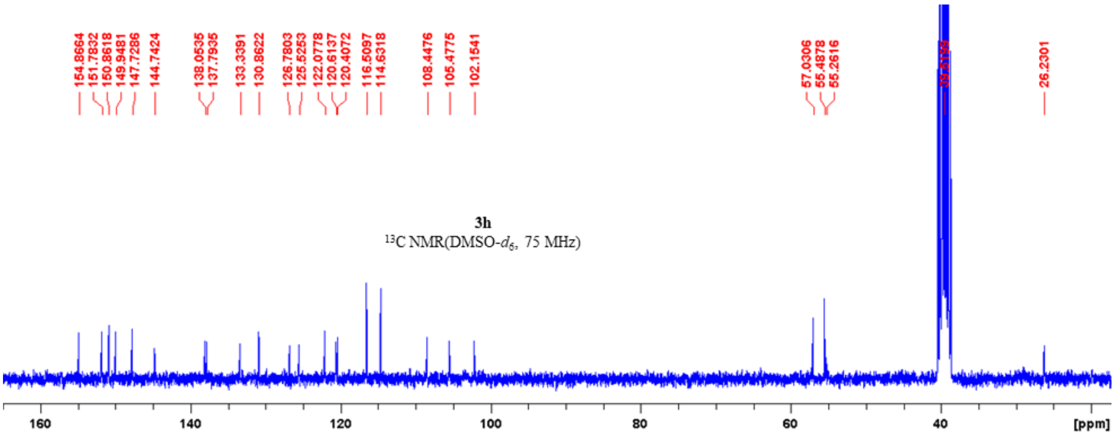
154.8664
151.7832
150.8618
149.9481
147.7286
144.7424
138.0535
137.7935
133.3391
130.8622
126.7803
125.5253
122.0778
120.6137
120.4072
116.5097
114.6318
108.4476
105.4775
102.1541
57.0306
55.4878
55.2616
26.2301
3h
13C NMR(DMSO-d6, 75 MHz)
160
140
120
100
80
60
40
[ppm]

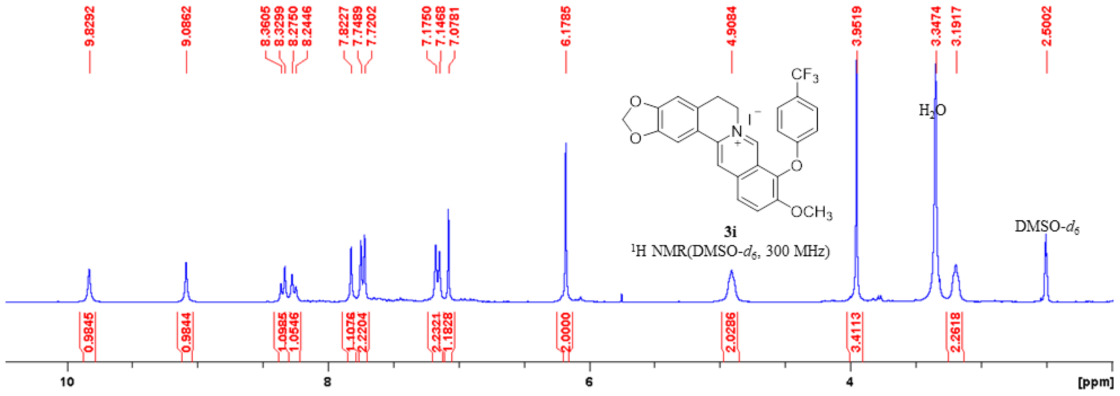

CF3
I⁻
O
OCH3
3i
1H NMR(DMSO-d6, 300 MHz)
H2O
DMSO-d6
[ppm]

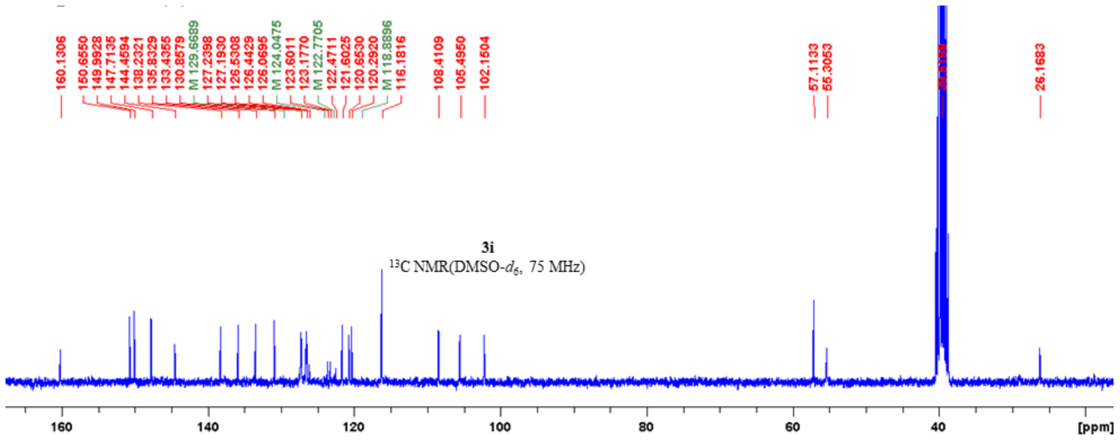

3i
13C NMR(DMSO-d6, 75 MHz)
[ppm]

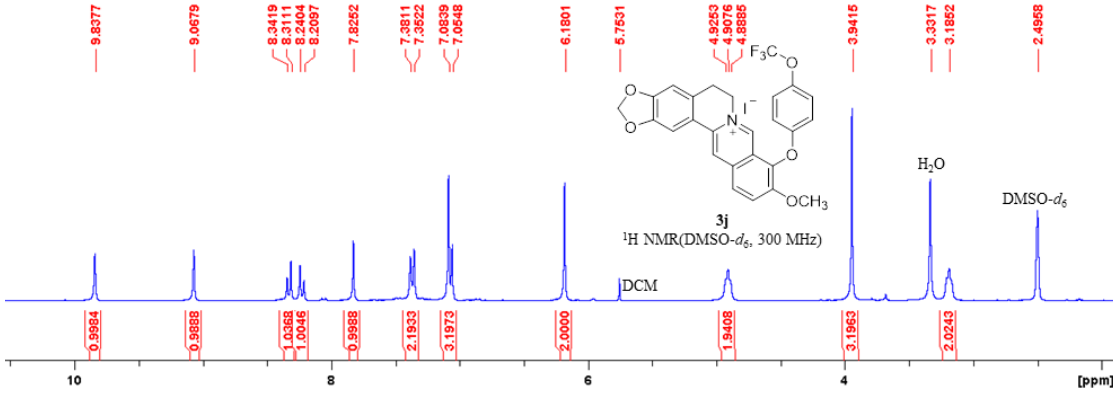

3j
1H NMR(DMSO-d6, 300 MHz)
H2O
DMSO-d6
DCM
[ppm]

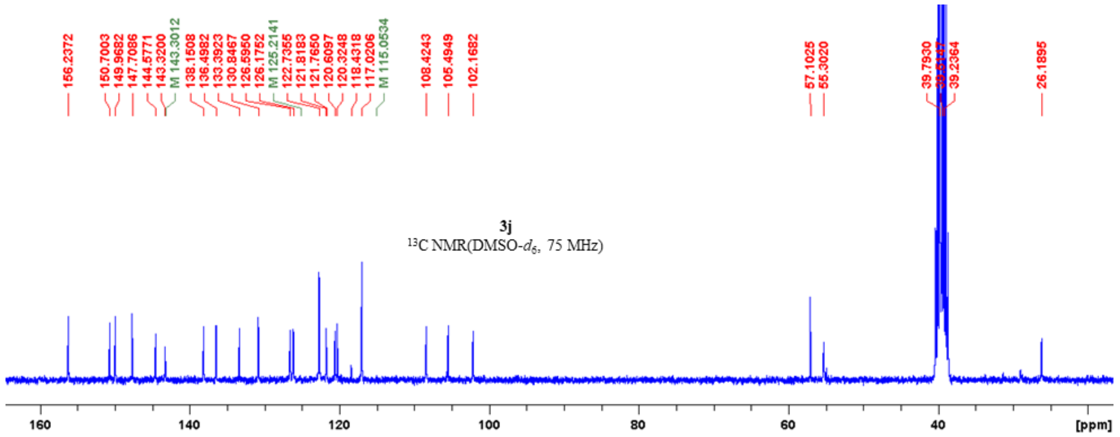

3j
13C NMR(DMSO-d6, 75 MHz)
[ppm]

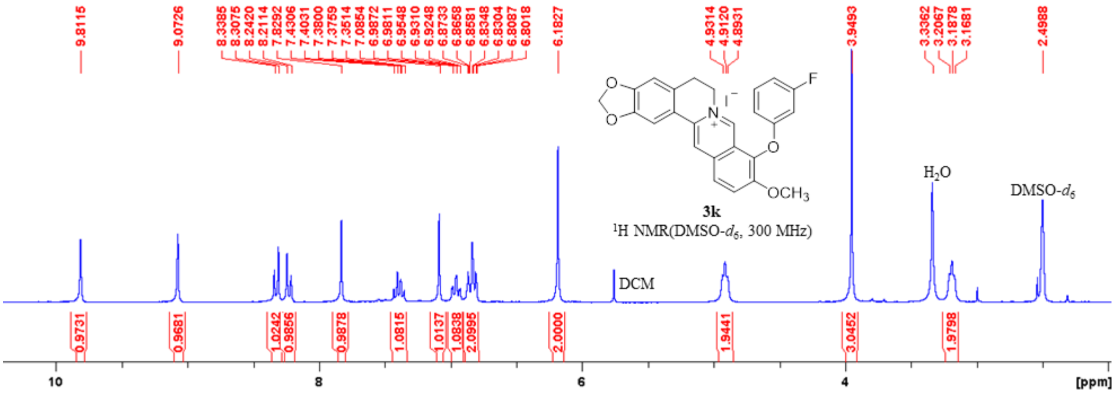

DCM
H2O
DMSO-d6
3k
1H NMR(DMSO-d6, 300 MHz)
[ppm]

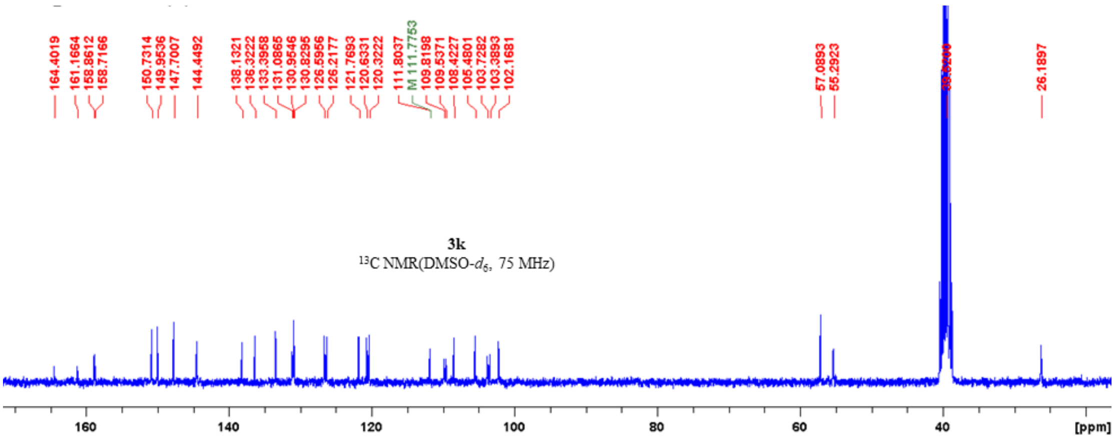

3k
13C NMR(DMSO-d6, 75 MHz)
[ppm]

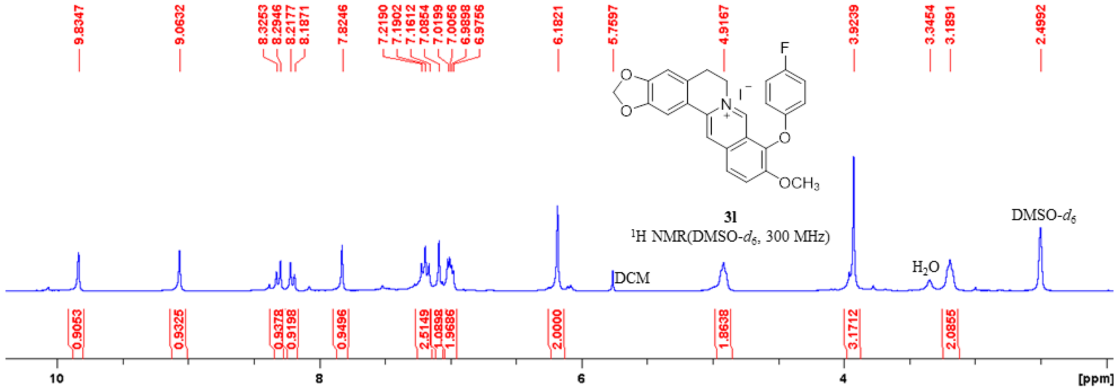
9.8347
9.0632
8.3253
8.2946
8.2177
8.1871
7.8246
7.2190
7.1902
7.1612
7.0854
7.0199
7.0056
6.9898
6.9756
6.1821
5.7597
4.9167
3.9239
3.3454
3.1891
2.4992
F
O
O
N
+
I
−
O
OCH3
3l
1H NMR(DMSO-d6, 300 MHz)
DCM
H2O
DMSO-d6
0.9053
0.9325
0.9378
0.9198
0.9496
2.5149
1.0898
1.9686
2.0000
1.8638
3.1712
2.0855
10
8
6
4
[ppm]

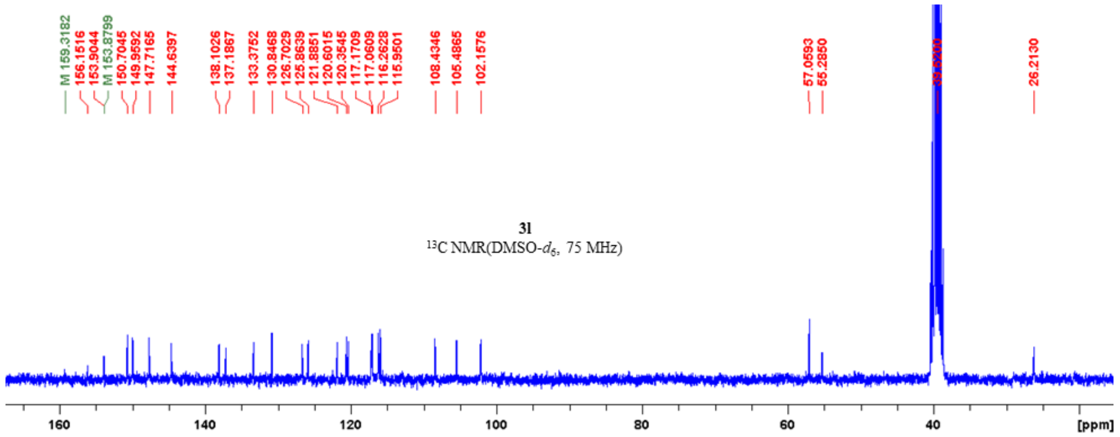
M 159.3182
156.1516
153.9044
M 153.8799
150.7045
149.9592
147.7165
144.6397
138.1026
137.1867
133.3752
130.8468
126.7029
125.8639
121.8851
120.6015
120.3545
117.1709
117.0609
116.2628
115.9501
108.4346
105.4865
102.1576
57.0593
55.2850
26.2130
3l
13C NMR(DMSO-d6, 75 MHz)
160
140
120
100
80
60
40
[ppm]

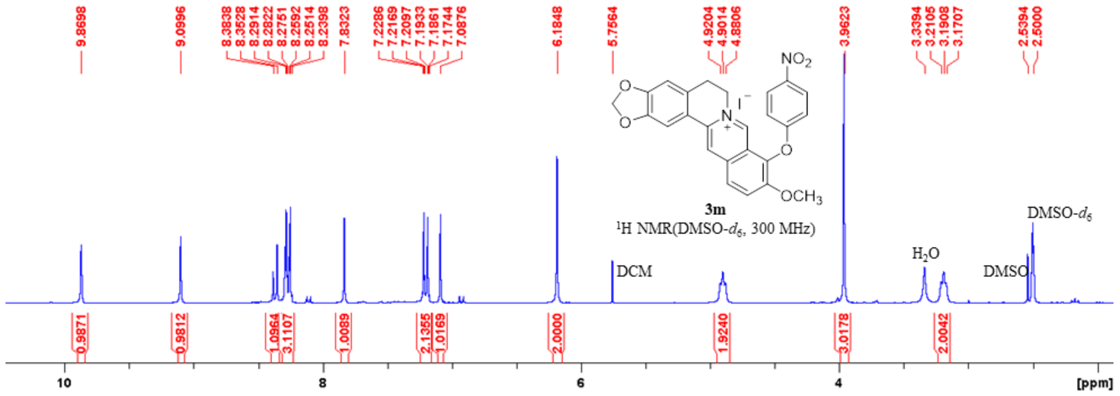

9.8698
9.0996
8.3838
8.3528
8.2914
8.2822
8.2751
8.2592
8.2514
8.2398
7.8323
7.2286
7.2169
7.2097
7.1933
7.1861
7.1744
7.0876
6.1848
5.7564
4.9204
4.9014
4.8806
3.9623
3.3394
3.2105
3.1908
3.1707
2.5394
2.5000
NO2
I−
N+
OCH3
3m
1H NMR(DMSO-d6, 300 MHz)
DCM
H2O
DMSO
DMSO-d6
0.9871
0.9812
1.0964
3.1107
1.0089
2.1355
1.0169
2.0000
1.9240
3.0178
2.0042
10
8
6
4
[ppm]

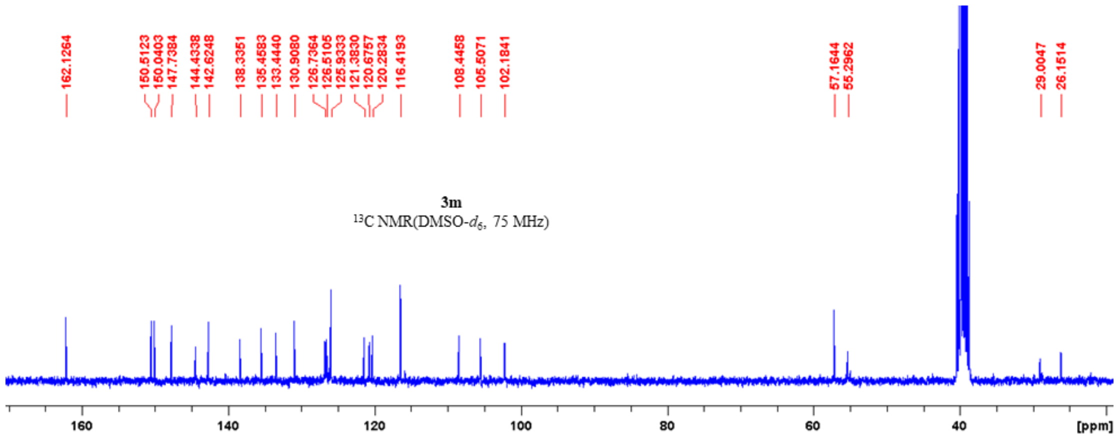

162.1264
150.5123
150.0403
147.7384
144.4338
142.6248
138.3351
135.4583
133.4440
130.9080
126.7364
126.5105
125.9333
121.3830
120.6757
120.2834
116.4193
108.4458
105.5071
102.1841
57.1644
55.2962
29.0047
26.1514
3m
13C NMR(DMSO-d6, 75 MHz)
160
140
120
100
80
60
40
[ppm]

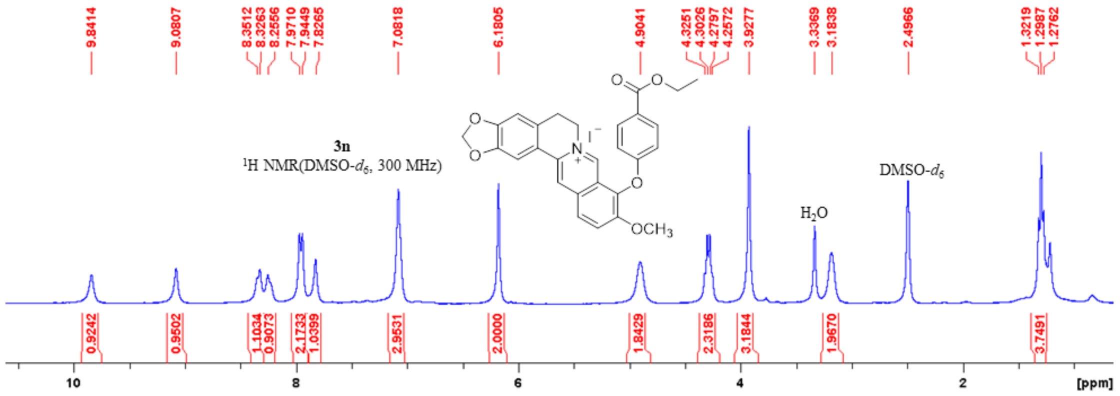

9.8414
9.0807
8.3512
8.3263
8.2556
7.9710
7.9449
7.8265
7.0818
6.1805
4.9041
4.3251
4.3026
4.2797
4.2572
3.9277
3.3369
3.1838
2.4966
1.3219
1.2987
1.2762
3n
¹H NMR(DMSO-d_6, 300 MHz)
I⁻
N⁺
OCH3
DMSO-d_6
H_2O
0.9242
0.9502
1.1034
0.9073
2.1733
1.0399
2.9531
2.0000
1.8429
2.3186
3.1844
1.9670
3.7491
10
8
6
4
2
[ppm]

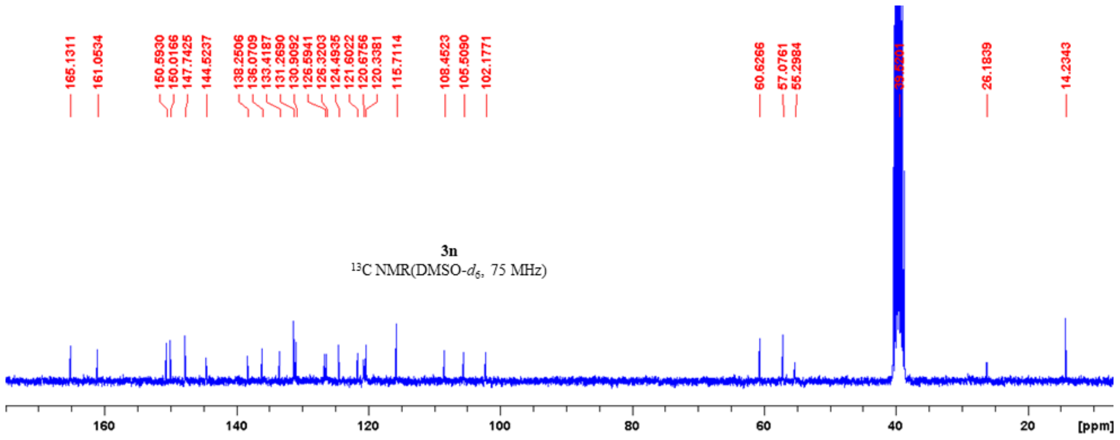

165.1311
161.0534
150.5930
150.0166
147.7425
144.5237
138.2506
136.0709
133.4187
131.2690
130.9092
126.5941
126.3203
124.4935
121.6022
120.6756
120.3381
115.7114
108.4523
105.5090
102.1771
60.6266
57.0761
55.2984
26.1839
14.2343
3n
¹³C NMR(DMSO-d_6, 75 MHz)
160
140
120
100
80
60
40
20
[ppm]

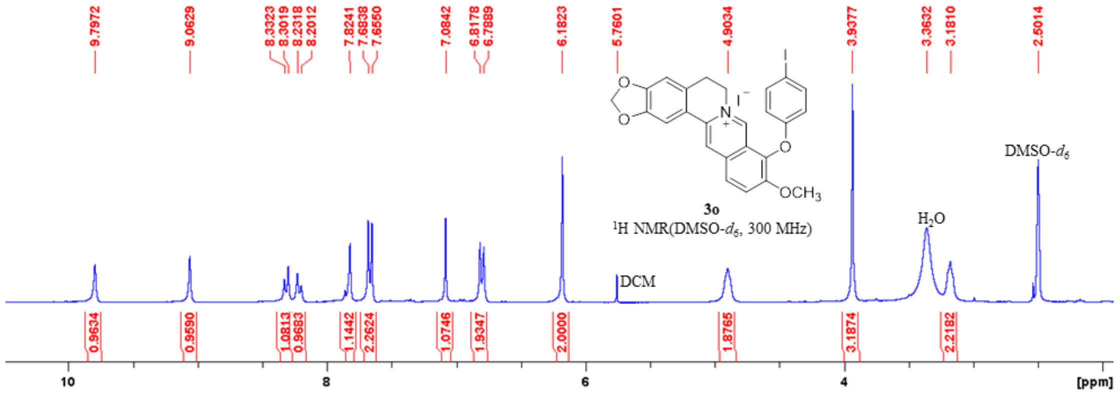
DCM
H2O
DMSO-d6
3o
1H NMR(DMSO-d6, 300 MHz)
[ppm]

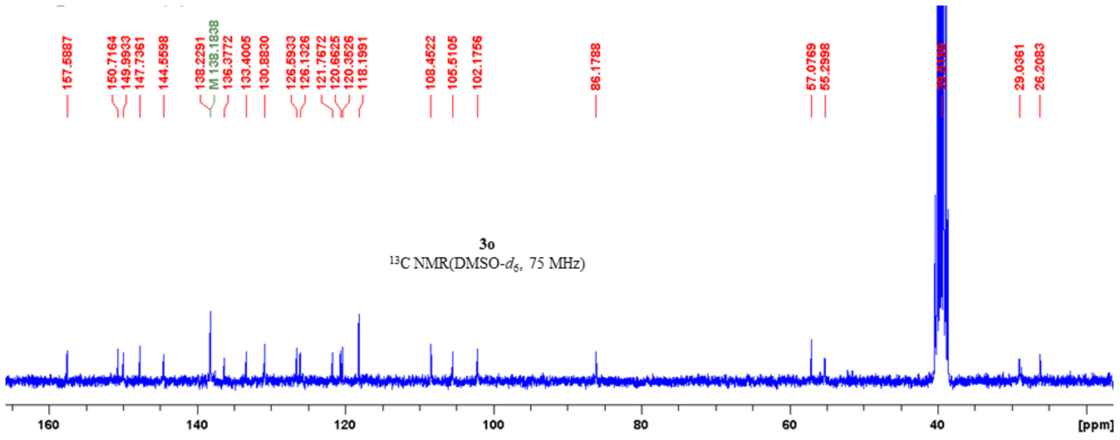
3o
13C NMR(DMSO-d6, 75 MHz)
[ppm]

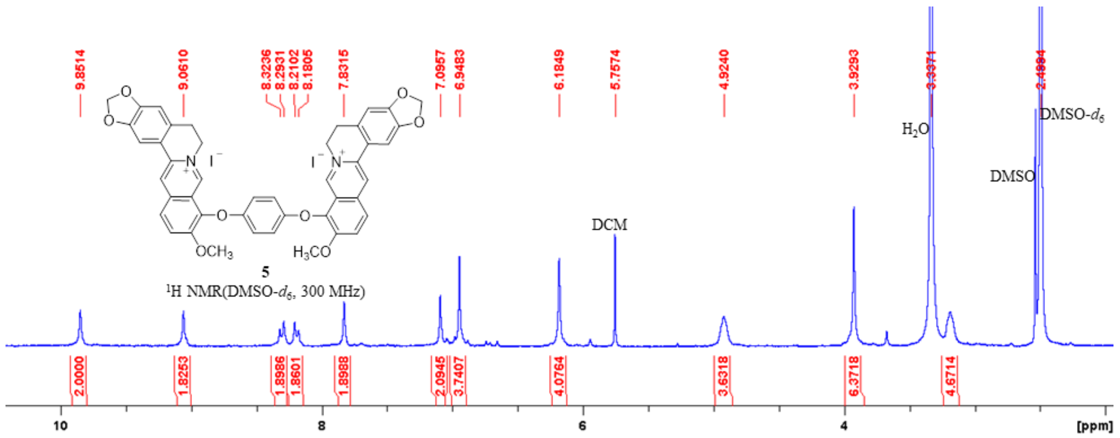
5
^1H NMR(DMSO-d_6, 300 MHz)
DCM
H_2O
DMSO
DMSO-d_6
9.8514
9.0610
8.3236
8.2931
8.2102
8.1805
7.8315
7.0957
6.9483
6.1849
5.7574
4.9240
3.9293
3.3371
2.4984
2.0000
1.8253
1.8986
1.8601
1.8988
2.0945
3.7407
4.0764
3.6318
6.3718
4.6714
10
8
6
4
[ppm]

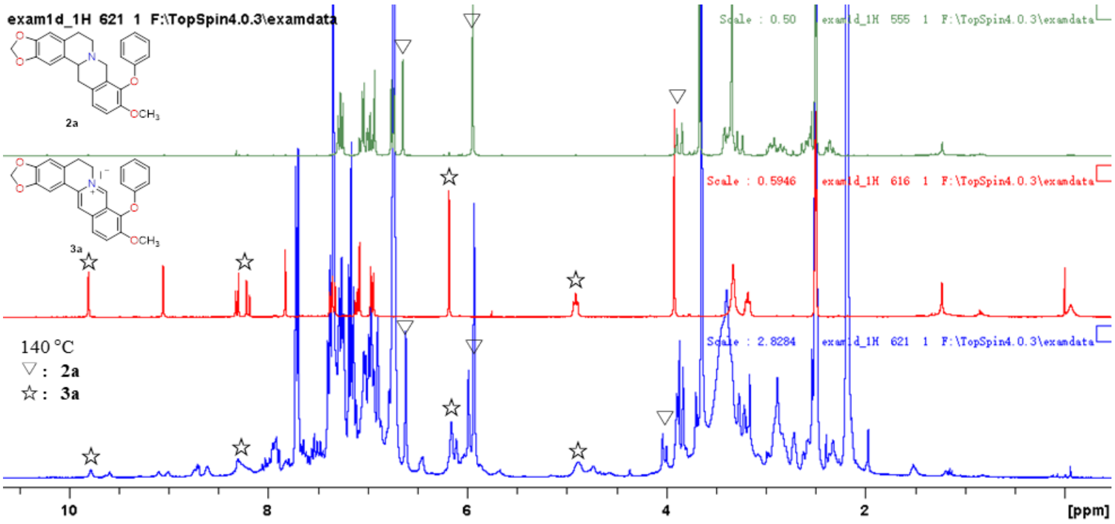

Figure S1: NMR analysis of reaction mixture at 140 °C.

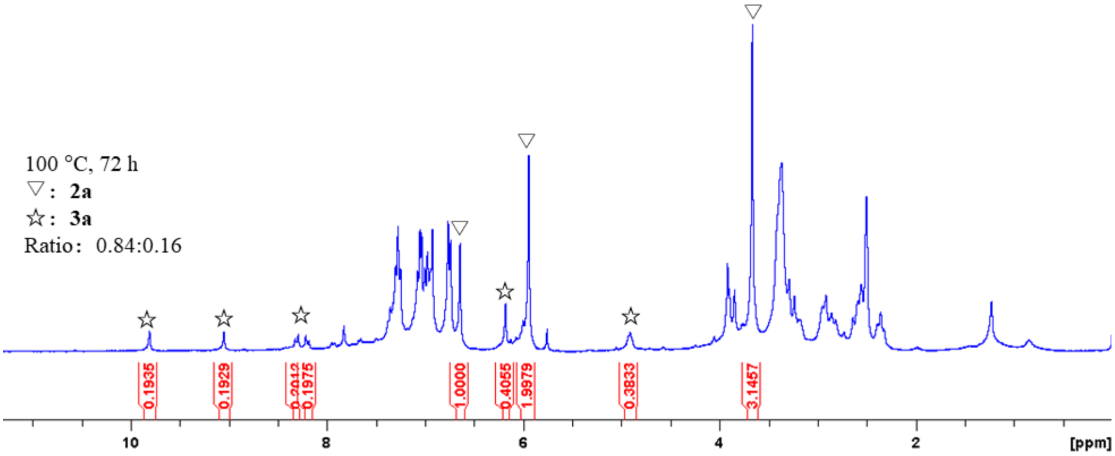

Figure S2: Oxidation of **2a** with DMSO.

Selected examples of HRMS spectra

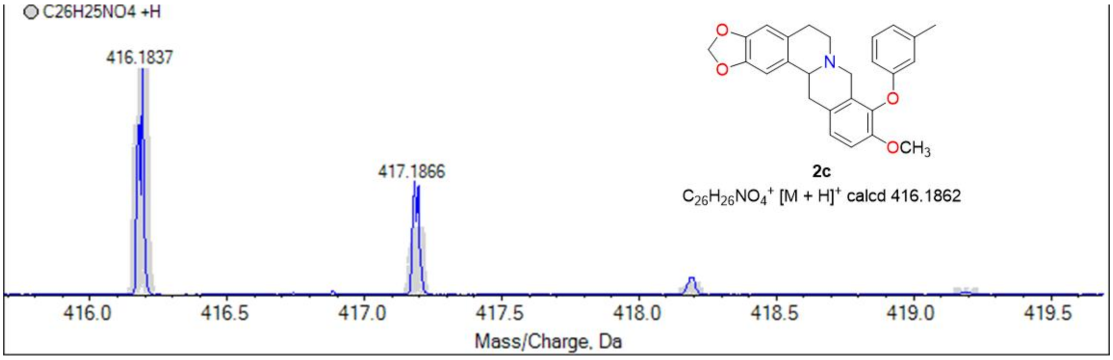

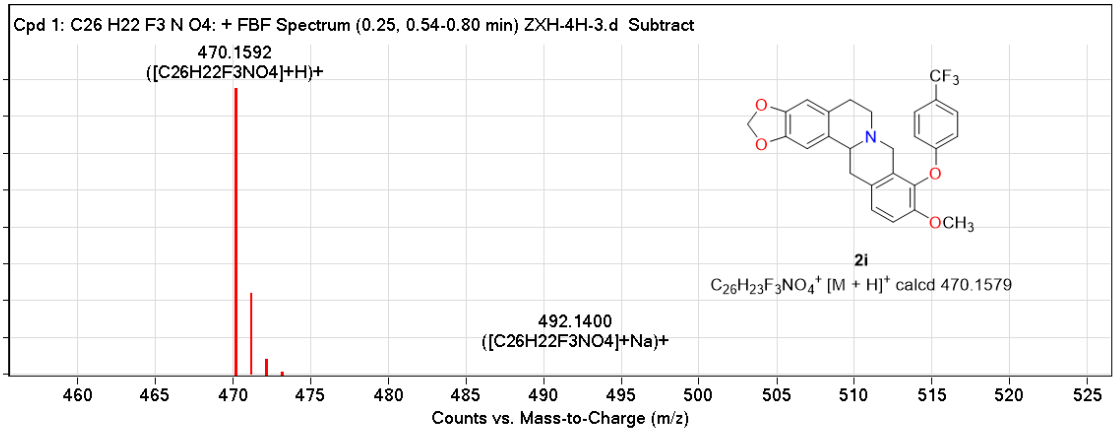

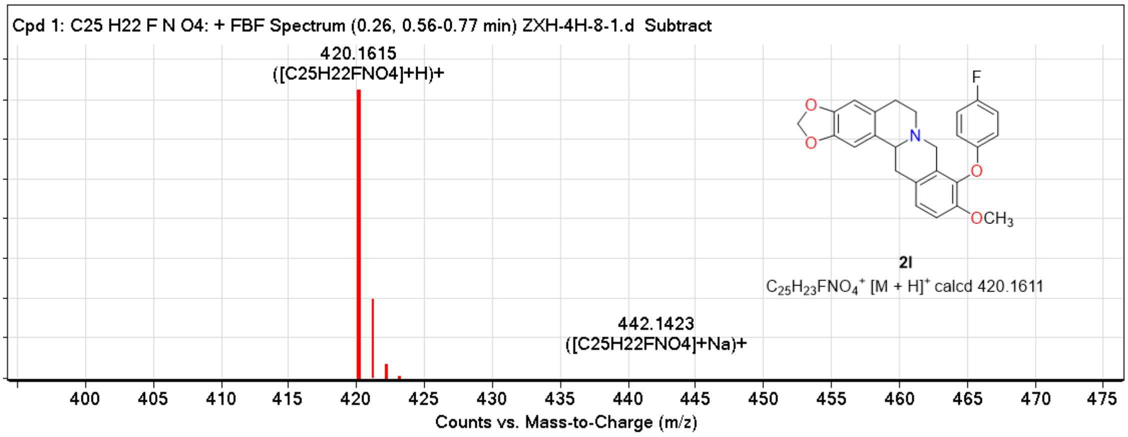

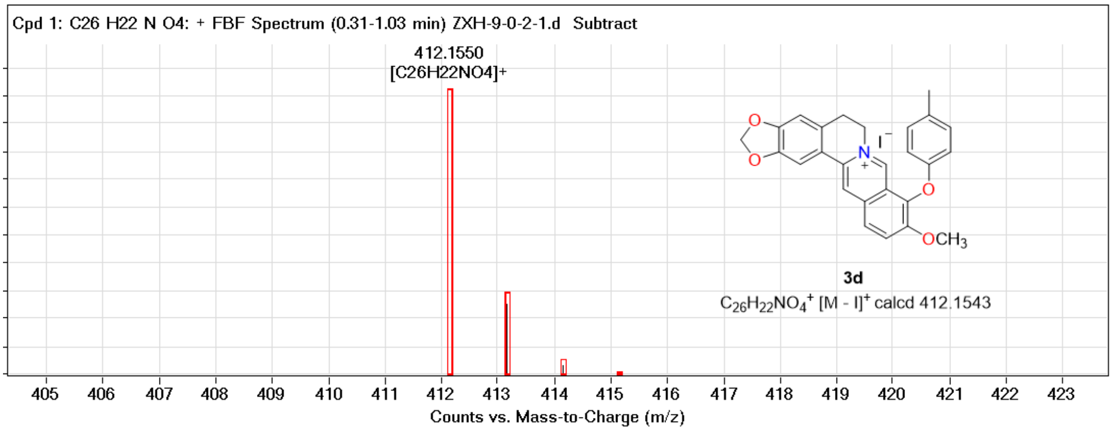
Cpd 1: C26 H22 N O4: + FBF Spectrum (0.31-1.03 min) ZXH-9-0-2-1.d Subtract
412.1550
[C26H22NO4]+
3d
$C_{26}H_{22}NO_4^+$ [M - I]$^+$ calcd 412.1543
405 406 407 408 409 410 411 412 413 414 415 416 417 418 419 420 421 422 423
Counts vs. Mass-to-Charge (m/z)

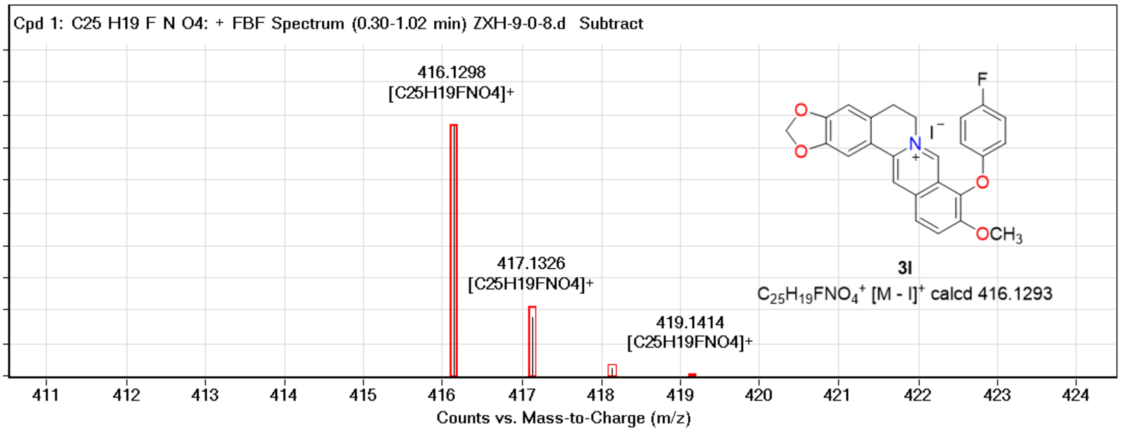
Cpd 1: C25 H19 F N O4: + FBF Spectrum (0.30-1.02 min) ZXH-9-0-8.d Subtract
416.1298
[C25H19FNO4]+
417.1326
[C25H19FNO4]+
419.1414
[C25H19FNO4]+
3l
$C_{25}H_{19}FNO_4^+$ [M - I]$^+$ calcd 416.1293
411 412 413 414 415 416 417 418 419 420 421 422 423 424
Counts vs. Mass-to-Charge (m/z)

Representative IR spectra

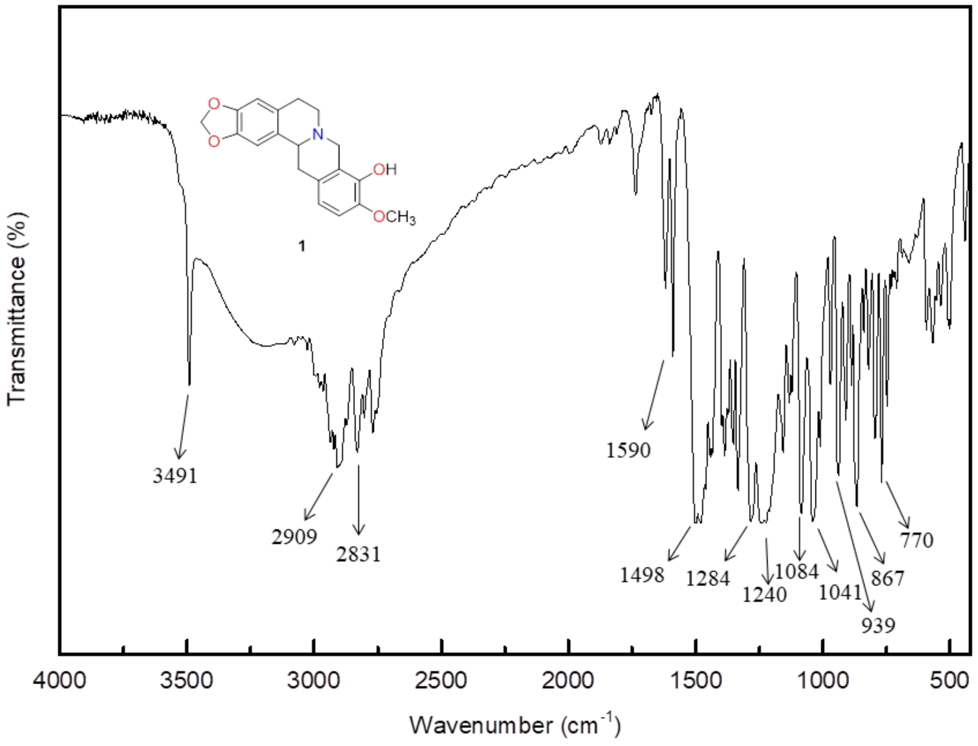

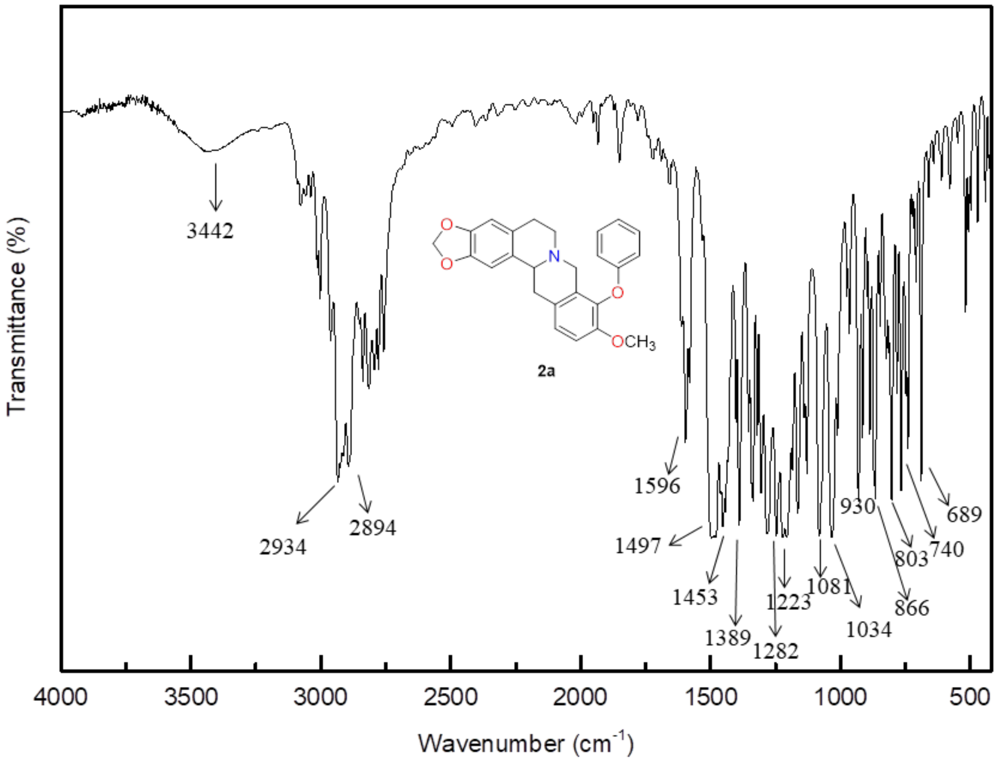

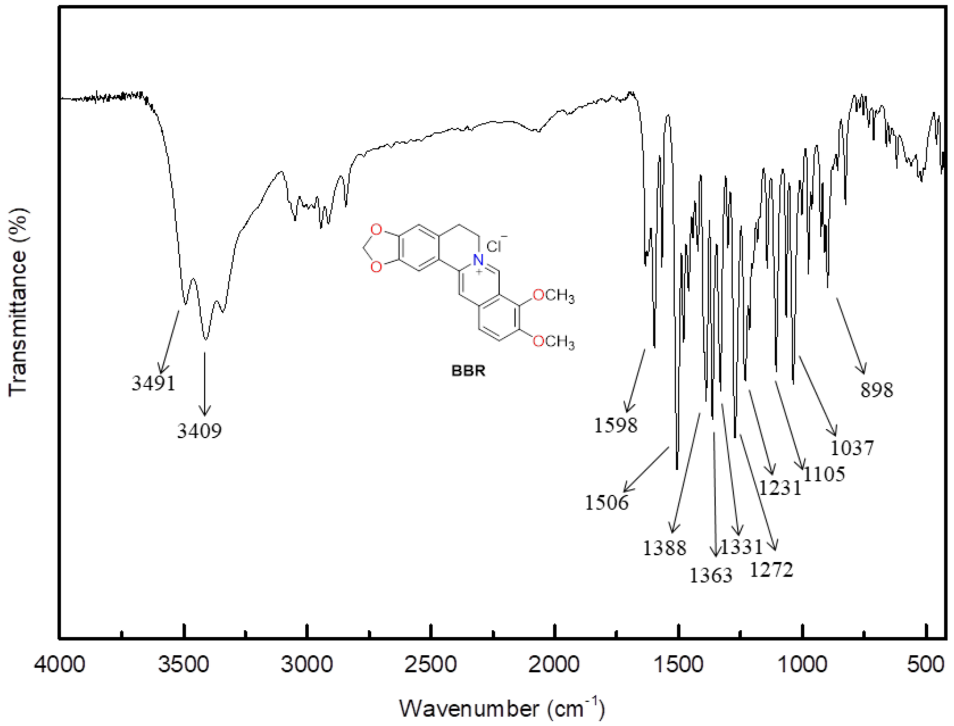

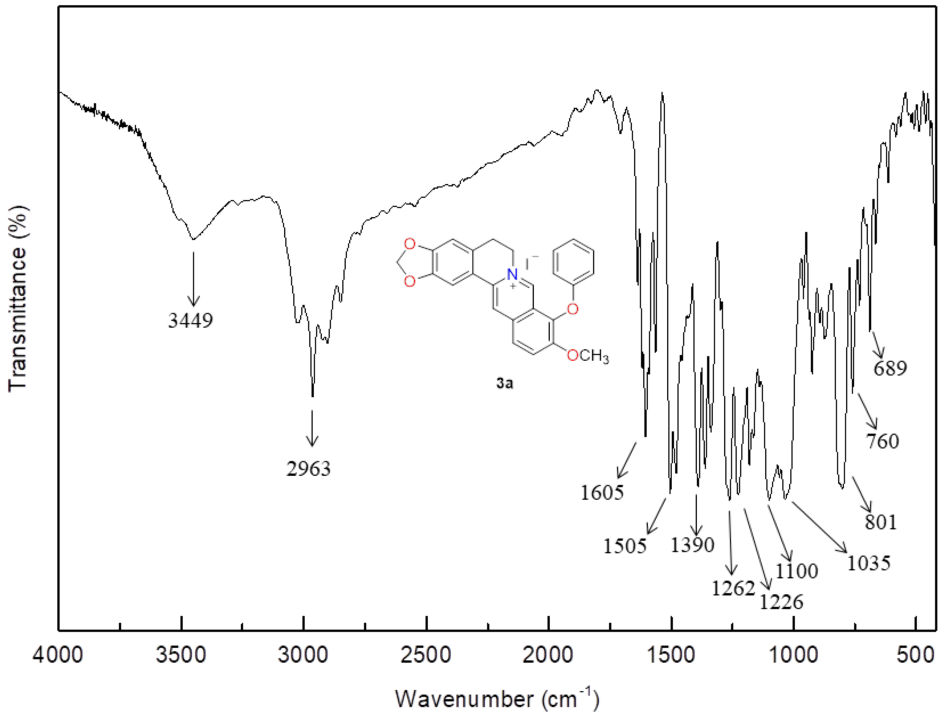

IR spectra discussion: The strong absorption bands in the range of 1200-1000 cm^{-1} are due to the C-O stretching vibrations. The absorptions at 740, 689 and 760, 689 cm^{-1} for

2a and **3a,** respectively, are assignable to the bending vibrations of the C-H bonds in mono-substituted phenyl ring.

References

1 Ge, H.-X.; Zhang, J.; Chen, L.; Kou, J.-P.; Yu, B.-Y. *Bioorg. Med. Chem.* **2013**, *21,* 62-69.
